# Design and Antiproliferative Evaluation of Novel Sulfanilamide Derivatives as Potential Tubulin Polymerization Inhibitors

**DOI:** 10.3390/molecules22091470

**Published:** 2017-09-05

**Authors:** Dong-Jun Fu, Ji-Feng Liu, Ruo-Han Zhao, Jia-Huan Li, Sai-Yang Zhang, Yan-Bing Zhang

**Affiliations:** 1 School of Pharmaceutical Sciences & Collaborative Innovation Center of New Drug Research and Safety Evaluation, Zhengzhou University, Zhengzhou 450001, China; zzufdj@sina.com (D.-J.F.); Liujf2009y@126.com (J.-F.L.); z13783586208@163.com (R.-H.Z.); lijiahuanzzu@163.com (J.-H.L.); 2 School of Basic Medical Sciences, Zhengzhou University, Zhengzhou 450001, China

**Keywords:** sulfanilamide-1,2,3-triazole, molecular hybridization strategy, antiproliferative activity, structure-activity relationships, tubulin polymerization

## Abstract

A series of sulfanilamide-1,2,3-triazole hybrids were designed by a molecular hybridization strategy and evaluated for antiproliferative activity against three selected cancer cell lines (MGC-803, MCF-7 and PC-3). The detailed structure-activity relationships for these sulfanilamide-1,2,3-triazole hybrids were investigated. All these sulfanilamide-1,2,3-triazole hybrids exhibited moderate to potent activity against all cell lines. In particular 4-methyl-*N*-((1-(3-phenoxybenzyl)-1*H*-1,2,3-triazol-4-yl)methyl)benzenesulfonamide (**11f**) showed the most potent inhibitory effect against PC-3 cells, with an IC_50_ value of 4.08 μM. Furthermore, the tubulin polymerization inhibitory activity in vitro of compound **11f** was 2.41 μM. These sulfanilamide hybrids might serve as bioactive fragments for developing more potent antiproliferative agents.

## 1. Introduction

**Figure 1:**
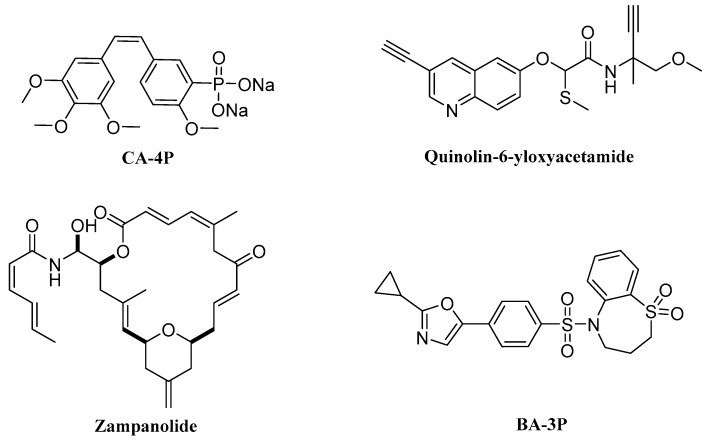
Potent tubulin-targeting agents.

Microtubules, as a key component of the cytoskeleton, play important roles in many cellular events, including the maintenance of cell shape, cell migration, cell division and intracellular transport [1,2,3,4]. Recently, many tubulin polymerization inhibitors were designed and synthesized. For example, combretastatin A-4P (**CA-4P**) was used as a clinical drug for the treatment of cancers [5]. Quinolin-6-yloxyacetamide (Figure 1) displayed potent antiproliferative activity against the A2780AD cell line, with an IC_50_ value of 262 nM by disrupting the microtubule cytoskeleton [6]. Zampanolide as a microtubule-stabilizing agent was active in resistant cancer cells and inhibited cell migration [7]. Benzenesulfonamide derivative **BA-3P** was designed as a potential anticancer agent with an IC_50_ value of 5.882 μM against PC3 cells targeting tubulin [8]. However, the major problem existing in clinical use of these anti-tubulin agents is their undesirable side effects, like neurotoxicity and cardiovascular toxicities [6,8]. Therefore, it is necessary to develop novel tubulin polymerization inhibitors for cancer therapy.

**Figure 2:**
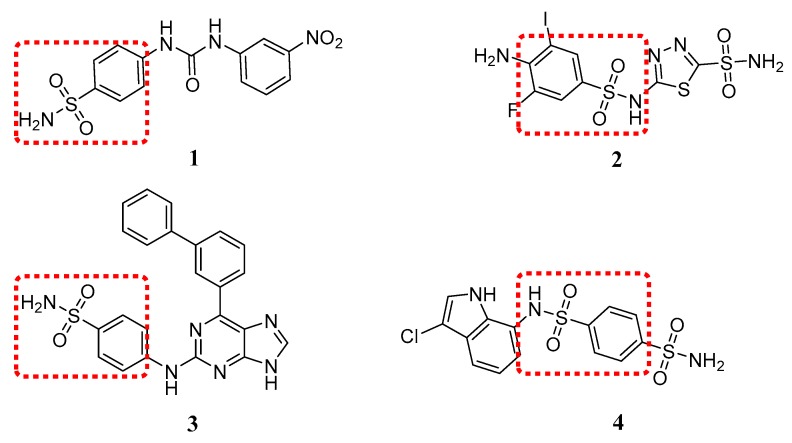
Antitumor sulfanilamides.

Sulfanilamide is a potential scaffold in antitumor drug discovery. Sulfanilamide derivative **1** (Figure 2) significantly inhibited the formation of metastases by the highly aggressive 4T1 mammary tumor cells at pharmacologic concentrations of 45 mg/kg [9]. 5-((4-Amino-3-fluoro-5-iodo-phenyl)sulfonamido)-1,3,4-thiadiazole-2-sulfonamide (**2**) was designed as a carbonic anhydrase inhibitor [10]. Benzenesulfonamide **3** as a selective cyclin-dependent kinase (CDK) inhibitor exhibited high potency toward CDK2 (IC_50_ 0.044 μM), but was ~2000-fold less active toward CDK1 (IC_50_ 86 μM) [11]. *N*-(3-chloro-7-indolyl)-1,4-benzenedisulfonamide **4** showed significant antitumor activity against HCT116 human colon carcinoma, with an IC_50_ value of 0.11 μg/mL in a cell proliferation assay [12].

**Figure 3:**
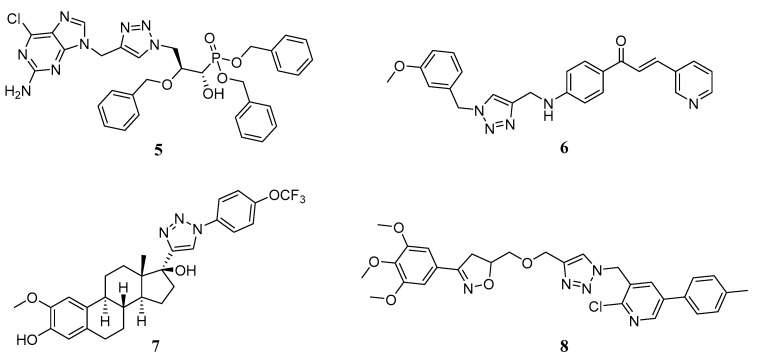
Antitumor 1,2,3-triazoles.

1,2,3-Triazoles were widely used as an anticancer scaffold in medicinal chemistry to design novel antitumor agents. (1*R*,2*S*)-1,2,3-triazole derivative **5** (Figure 3) displayed potent inhibitory effects against the proliferation of murine leukemia with an IC_50_ value of 21 μM [13]. Chalcone-1,2,3-triazole **6** designed in our group could inhibit the proliferation of SK-N-SH cancer cells by inducing apoptosis and arresting the cell cycle at the G1 phase [14]. On the other hand, the 1,2,3-triazole moiety has also been used as an important linker to design novel tubulin polymerization inhibitors. For example, the 1,4-disubstituted 1,2,3-triazole analog **7** based on 2-methoxyestradiol exhibited anti-proliferative effects at low micromolar concentrations and inhibited tubulin assembly with an IC_50_ value of 5.9 μM [15]. Pyridinyl-1*H*-1,2,3-triazolyldihydroisoxazole **8** was designed as a tubulin polymerization inhibitor with a concomitant accumulation of cells in the G2/M phase of the cell cycle [16]. 

**Figure 4:**
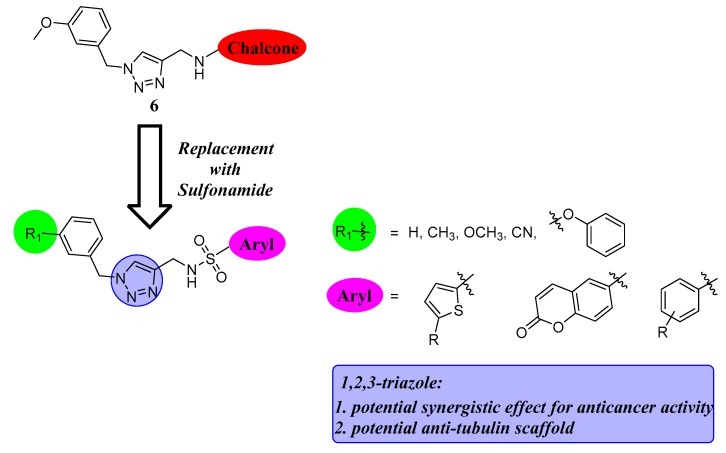
Rational design strategy for novel sulfonamide-1,2,3-triazoles.

These interesting findings and our continuous quest to identify more potent anticancer agents, led us to use the molecular hybridization of 1,2,3-triazole and sulfonamide groups to generate a novel hybrid with potent antiproliferative activity. As shown in Figure 4, the novel hybrid based on the structures of 1,2,3-triazole and sulfonamide has three parts: an aryl sulfonamide (thiophenesulfonamide, coumarin sulfonamide, benzenesulfonamide), the potential anti-tubulin 1,2,3-triazole scaffold and various phenyl units. Importantly, detailed structure activity relationships (*SARs*) of these three regions were investigated in this work. These novel sulfonamide-1,2,3-triazole hybrids might serve as bioactive fragments and lead compounds for developing more potent cytotoxic agents and tubulin polymerization inhibitors.

## 2. Results and Discussion

### 2.1. Chemistry

**Scheme 1:**
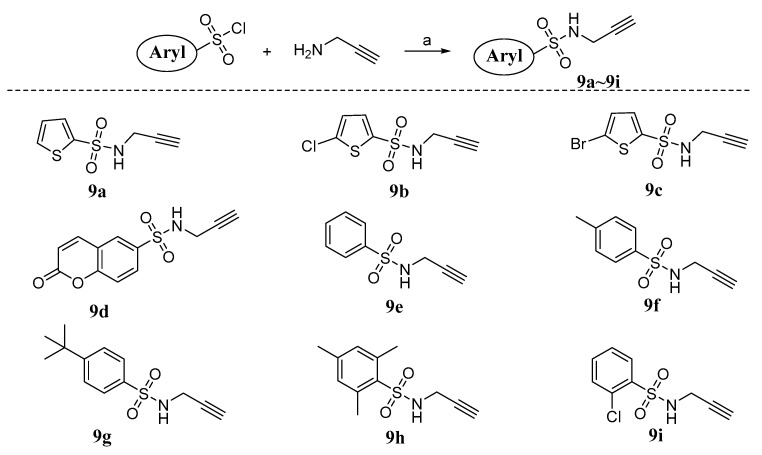
Synthesis of alkyne intermediates **9a~i**. *Reagents and conditions*: (a) K_2_CO_3_, dichloromethane, r.t.

**Figure 5:**
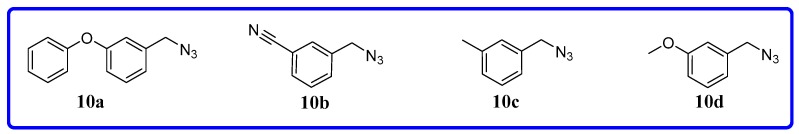
Azides **10a~d** in the present study.

Alkyne intermediates **9a~i** were synthesized as shown in Scheme 1. Commercially available aryl sulfonyl chlorides (thiophenesulfonyl chloride, coumarinsulfonyl chloride, benzenesulfonyl chloride) were treated with propynylamine in the presence of potassium carbonate to provide **9a~i**. Azide derivatives **10a~d** used in this work (Figure 5) were purchased from Zhengzhou Research Biotechnology Co., Ltd. (Zhengzhou, China).

**Scheme 2:**
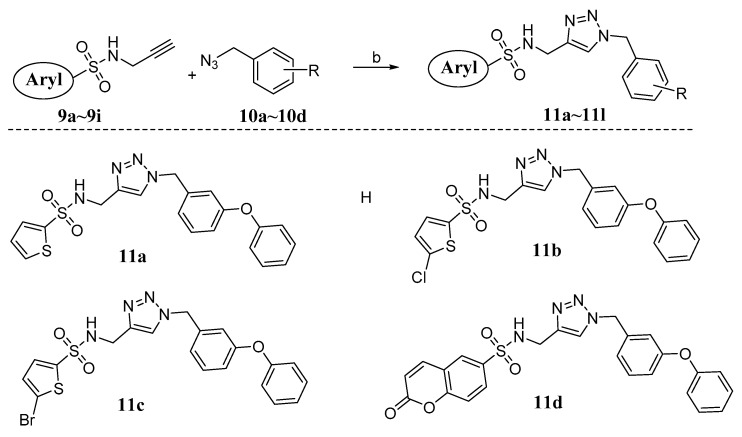

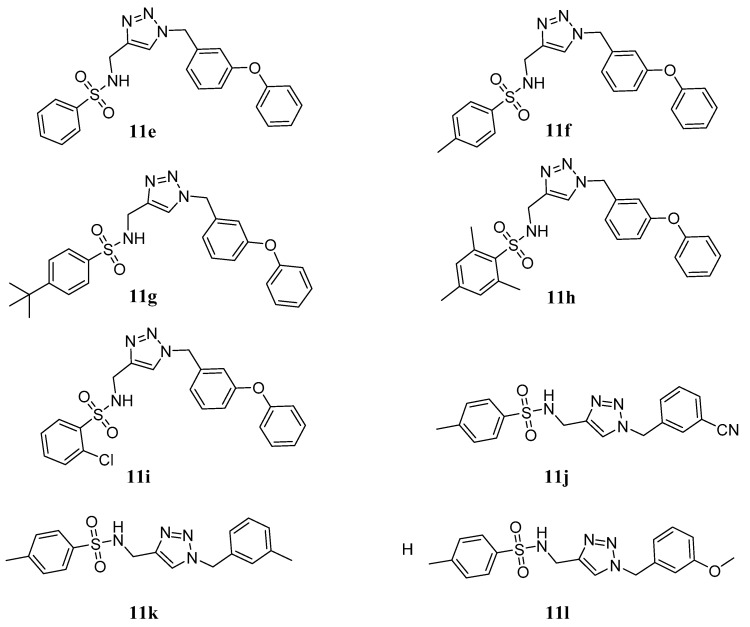
Synthesis of target hybrids **11a~l**. *Reagents and conditions*: (b) CuSO_4_·5H_2_O, sodium ascorbate, THF:H_2_O (1:1), r.t.

The applications of click chemistry are increasingly found in all aspects of drug discovery, ranging from lead discovery through combinatorial chemistry and target-templated in situ chemistry, to proteomics and DNA research, using bioconjugation reactions [17,18,19,20]. The sulfonamide-1,2,3-triazole hybrids **11a~l** were synthesized through a click reaction between alkyne intermediates **9a~i** and derivatives **10a~d** using copper sulfate and sodium ascorbate in THF/H_2_0 (Scheme 2).

### 2.2. Antiproliferative Activity and SARs Analysis

All synthesized sulfonamide-1,2,3-triazole hybrids were evaluated for their anticancer activity against three cancer cell lines: MGC-803 (human gastric cancer cell line), MCF-7 (human breast cancer cell line), and PC-3 (human prostate cancer cell line) using a MTT (4,5-dimethyl-2-thiazolyl)-2,5-diphenyl-2-*H*-tetrazolium bromide) assay [21,22,23]. The well-known tubulin polymerization inhibitor **CA-4P** was used as a control. In [24], 5-fluorouracil (**5-Fu**) was selected as the control in the antiproliferative evaluation assay of 1,2,3-triazole derivatives. Therefore, **5-Fu** was also used as the reference drug in this MTT assay.

The antiproliferative results are summarized in Table 1. In order to investigate the effect of the inhibitory activity of 1,2,3-triazole moiety, the antiproliferative activity of the non-1,2,3-triazole-sulfonamide derivatives **9a**, **9d** and **9f** and the 1,2,3-triazole-sulfonamide hybrids **11a~l** was investigated. Removal of the 1,2,3-triazole skeleton was clearly detrimental for the inhibitory activity against the three cancer cell lines, as shown by the results of compounds **9a**, **9d** and **9f**. The introduction of 1,2,3-triazole scaffold resulted in a significant improvement of inhibitory activity, suggesting that the 1,2,3-triazole moiety displays a synergistic role in determining activity. In particular, compound **11f** showed more potent inhibitory effects against three cell lines than **5-Fu**, with IC_50_ values ranging from 4.1 μM to 15.7 μM. 

In order to complete the structure activity relationship study, compounds with a thiophene ring (**11a~c**), a coumarin ring (**11d**), a phenyl ring (**11i**), a formononetin scaffold (**11j**) and coumarin scaffolds (**11e~h**) were synthesized to explore the effect of aryl rings for their antiproliferative activity. Among all the thiophenesulfonamide derivatives **11a~c**, compound **11b** with a 4-Cl-thiophene ring displayed the most potent antiproliferative activity against three selected cell lines. Coumarin-1,2,3-triazole-sulfonamide hybrid **11d** showed weak antiproliferative activity with IC_50_ values ranging from 34.1 μM to 88.8 μM. These results suggested that heteroaromatic rings play an important role in the inhibitory activity.

We also found that the phenyl ring was important for the activity, shown by an improvement for inhibitory activity against PC-3 cells, when the thiophene ring and coumarin ring were replaced with a phenyl ring (compounds **11a~d** vs. **11e**). In addition, the substituent on the phenyl ring bearing the sulfonamide may affected the antiproliferative activity. Replacement of the hydrogen atom of compound **11e** with a tertiary butyl group as in compound **11g** led to a decrease of the activity. However, changing the 2,4,6-triCH_3_ substitution pattern (compound **11h**) and 2-Cl (compound **11i**) to 4-CH_3_ (compound **11f**) led to an improvement of activity against all three cell lines, indicating the significance of the 4-CH_3_ group on the phenyl ring attaching sulfonamide in their antiproliferative activity.

Furthermore, a phenoxy group on the phenyl attaching the 1,2,3-triazole played a key role in the antiproliferative activity. Removal of the phenoxy group (**11f**) was clearly detrimental for the inhibitory activity against the three cancer cell lines, as shown by compounds **11j~l** without a phenoxy group which displayed no inhibitory activity against MCF-7 cells and PC-3 cells, with IC_50_ values of >100 μM.

### 2.3. In Vitro Tubulin Polymerization Inhibitory Activity Assay

**Figure 6:**
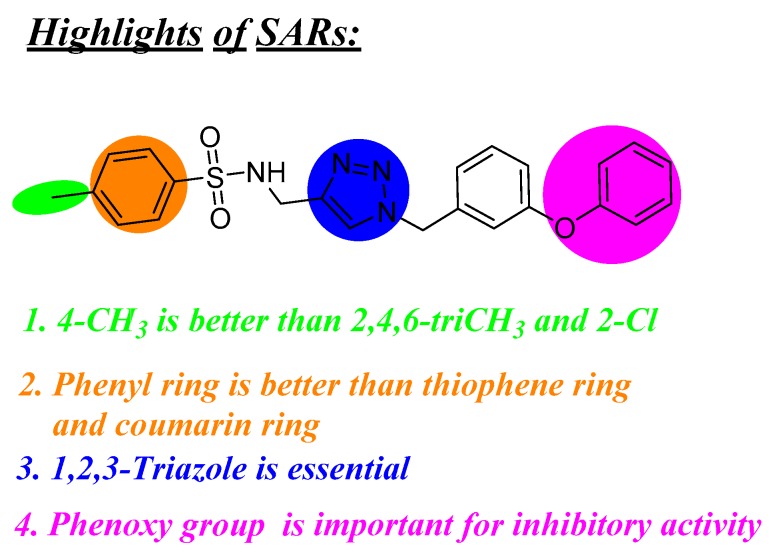
Summary of *SARs*.

Compound **11f** was further examined for possible inhibitory effects against GES-1 (normal human gastric epithelial cell line). We found that compound **11f** exhibited no significantly inhibitory effect against GES-1 (>64 μM). This fact, combined with its more potent antiproliferative activity against the selected MGC-803 cancer cell line than **5-Fu**, with an IC_50_ value of 13.7 μM, indicated that compound **11f** had good selectivity between cancer and normal cells. An illustration summarizing the structure activity relationships of the target derivatives in provided in Figure 6.

**Figure 7:**
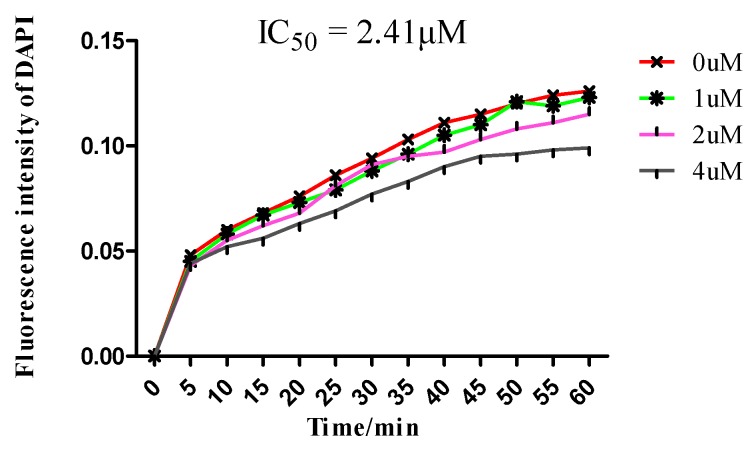
Tubulin polymerization inhibitory activity of compound **11f**.

The inhibitory concentrations that reduced the polymerized tubulin by 50% of **CA-4P** was 2.40 μM [25]. To investigate whether the synthesized 1,2,3-triazole-sulfonamide hybrids target the tubulin-microtubule system, the in vitro tubulin polymerization inhibition activity of compound **11f** was evaluated because of its best antiproliferative activity results among all the tested compounds in the initial cytotoxicity screening. When tubulin was incubated with the tested 1,2,3-triazole-sulfonamide **11f** at the indicated concentrations, an increased fluorescence intensity was obvious. The inhibitory concentrations that reduced the polymerized tubulin by 50% (IC_50_) of compound **11f** was 2.41 μM (Figure 7). This revealed **11f** was a novel tubulin polymerization inhibitor. 

## 3. Materials and Methods 

### 3.1. General Chemical Experimental Procedures

All reagents and solvents were of analytical grade and purchased from commercial sources. Thin-layer chromatography was carried out on glass plates coated with silica gel and visualized by UV light (254 nm). All NMR spectra were recorded with a DPX 400 MHz spectrometer (Bruker, Switzerland). Mass spectra (MS) were recorded on an Esquire 3000 mass spectrometer by electrospray ionization (ESI) (Micromass UK Limited, Manchester, England).

#### 3.1.1. General Procedure for the Synthesis of Compounds **9a~i**

To a stirred solution of the appropriate sulfonyl chloride (5 mmol) in dichloromethane (20 mL), propargylamine (5 mmol) and K_2_CO_3_ (5 mmol) were added carefully and the reaction mixture was stirred at room temperature for 6 h. The system was dissolved in dichloromethane (20 mL) and washed with water, brine, dried over anhydrous Na_2_SO_4_ and concentrated under vacuum to afford compounds **9a~i**, which were used in the next reaction without further purification. Copies of ^1^H- and ^13^C-NMR spectras for compounds **9a~i** were shown in supplementary materials.

*N-(Prop-2-yn-1-yl)thiophene-2-sulfonamide* (**9a**). Yellow Liquid, yield: 49.2%. ^1^H-NMR (400 MHz, CDCl_3_) δ 7.59 (dt, *J* = 9.3, 4.7 Hz, 1H, Ar), 7.55 (dd, *J* = 5.0, 1.3 Hz, 1H, Ar), 7.03 (dd, *J* = 5.0, 3.8 Hz, 1H, Ar), 5.26 (s, 1H, NH), 3.83 (dd, *J* = 6.0, 2.5 Hz, 2H, CH_2_), 2.12–2.03 (m, 1H, CCH). ^13^C-NMR (100 MHz, CDCl_3_) δ 139.33, 131.82, 131.33, 126.44 (Ar), 76.69, 71.91 (CCH), 32.05 (CH_2_). HRMS (ESI) calcd. for C_7_H_8_NO_2_S_2_ [M + H]^+^: 201.9999, found: 201.9996. IR: 3264, 1426, 1327, 1161, 1067, 878, 715, 666, 595 cm^−1^.

*5-Chloro-N-(prop-2-yn-1-yl)thiophene-2-sulfonamide* (**9b**). Orange liquid, yield: 67.9%. ^1^H-NMR (CDCl_3_) δ 7.39 (d, *J* = 4.0 Hz, 1H, Ar), 6.87 (d, *J* = 4.0 Hz, 1H, Ar), 5.09 (s, 1H, NH), 3.84 (dd, *J* = 5.6, 2.4 Hz, 2H, CH_2_), 2.12 (dd, *J* = 5.1, 2.6 Hz, 1H, CCH). ^13^C-NMR (CDCl_3_) δ 137.23, 136.79, 131.32, 125.79 (Ar), 76.41, 72.26 (CCH), 32.04 (CH_2_). HRMS (ESI) calcd. for C_7_H_7_ClNO_2_S_2_ [M + H]^+^: 235.9608, found: 235.9607. IR: 3273, 1413, 1330, 1152, 1084, 994, 801, 647, 616 cm^−1^.

*5-Bromo-N-(prop-2-yn-1-yl)thiophene-2-sulfonamide* (**9c**). Orange liquid, yield: 45.0%. ^1^H-NMR (CDCl_3_) δ 7.35 (d, *J* = 4.0 Hz, 1H, Ar), 7.01 (dd, *J* = 3.9, 1.8 Hz, 1H, Ar), 4.93 (d, *J* = 77.0 Hz, 1H, NH), 3.84 (d, *J* = 2.1 Hz, 2H, CH_2_), 2.13 (t, *J* = 2.5 Hz, 1H, CCH). ^13^C-NMR (CDCl_3_) δ 140.12, 132.00, 129.42, 119.47 (Ar), 72.32, 72.27 (CCH), 32.08 (CH_2_). HRMS (ESI) calcd. for C_7_H_7_BrNO_2_S_2_ [M + H]^+^: 279.9107, found: 279.9102. IR: 3303, 3266, 1428, 1401, 1354, 1329, 1156, 1060, 969, 805, 676, 629, 572 cm^−1^.

*2-Oxo-N-(prop-2-yn-1-yl)-2H-chromene-6-sulfonamide* (**9d**). White solid, yield: 20.7%, m.p.: 193–196 °C. ^1^H-NMR (DMSO-*d*_6_) δ 8.25 (dt, *J* = 12.8, 7.8 Hz, 3H, Ar), 7.99 (dd, *J* = 8.7, 2.2 Hz, 1H, Ar), 7.60 (d, *J* = 8.7 Hz, 1H, Ar), 6.64 (d, *J* = 9.6 Hz, 1H, NH), 3.86–3.65 (m, 2H, CH_2_), 3.02 (t, *J* = 2.5 Hz, 1H, CCH). ^13^C-NMR (CDCl_3_) δ 164.53, 160.92, 148.91, 141.78, 135.21, 133.00, 132.91, 123.97, 122.79, 122.67 (Ar), 84.38, 80.17 (CCH), 37.14 (CH_2_). HRMS (ESI) calcd. for C_12_H_10_NO_4_S [M + H]^+^: 264.0335, found: 264.0331. IR: 3287, 3216, 1703, 1598, 1336, 1161, 1116, 1074, 834, 675, 598 cm^−1^.

*N-(Prop-2-yn-1-yl)benzenesulfonamide* (**9e**). Colorless liquid, yield: 93.8%. ^1^H-NMR (CDCl_3_) δ 8.02–7.73 (m, 2H, Ar), 7.65–7.34 (m, 3H, Ar), 5.08 (s, 1H, NH), 3.77 (dd, *J* = 6.1, 2.5 Hz, 2H, CH_2_), 2.00 (t, *J* = 2.5 Hz, 1H, CCH). ^13^C-NMR (CDCl_3_) δ 138.50, 131.97, 128.08, 126.33 (Ar), 76.90, 71.98 (CCH), 31.80 (CH_2_). HRMS (ESI) calcd. for C_9_H_10_NO_2_S [M + H]^+^: 196.0434, found: 196.0432. IR: 3278, 3254, 1445, 1166, 1093, 1067, 759, 723, 657, 597 cm^−1^.

*4-Methyl-N-(prop-2-yn-1-yl)benzenesulfonamide* (**9f**). White solid, yield: 41.4%, m.p.: 70–75 °C. ^1^H-NMR (CDCl_3_) δ 7.71 (d, *J* = 8.1 Hz, 2H, Ar), 7.23 (t, *J* = 10.6 Hz, 2H, Ar), 4.66 (s, 1H, NH), 3.76 (dd, *J* = 5.9, 2.4 Hz, 2H, CH_2_), 2.36 (s, 3H, CH_3_), 2.03 (t, *J* = 2.2 Hz, 1H, CCH). ^13^C-NMR (CDCl_3_) δ 142.85, 135.51, 128.70, 126.39 (Ar), 76.94, 71.98 (CCH), 31.85 (CH_2_), 20.54 (CH_3_). HRMS (ESI) calcd. for C_10_H_12_NO_2_S [M + H]^+^: 210.0588, found: 210.0589. IR: 3273, 1325, 1158, 1069, 703, 669, 576, 547 cm^−1^.

*4-(Tert-butyl)-N-(prop-2-yn-1-yl)benzenesulfonamide* (**9g**). White solid, yield: 30.9%, m.p.: 58–63 °C. ^1^H-NMR (CDCl_3_) δ 7.74 (d, *J* = 8.5 Hz, 2H, Ar), 7.46 (d, *J* = 8.5 Hz, 2H, Ar), 4.68 (s, 1H, NH), 3.76 (d, *J* = 2.4 Hz, 2H, CH_2_), 2.02 (s, 1H, CCH), 1.27 (s, 9H, C(CH_3_)_3_). ^13^C-NMR (CDCl_3_) δ 155.90, 135.31, 126.21, 125.09 (Ar), 76.96, 71.91 (CCH), 34.16 (*C*(CH_3_)_3_), 31.90 (CH_2_), 30.06 (C(*C*H_3_)_3_). HRMS (ESI) calcd. for C_13_H_18_NO_2_S [M + H]^+^: 252.1059, found: 252.1058. IR: 3314, 3266, 2961, 2869, 1596, 1327, 1162, 1112, 822, 636, 570, 539 cm^−1^.

*2,4,6-Trimethyl-N-(prop-2-yn-1-yl)benzenesulfonamide* (**9h**). White solid, yield: 74.2%, m.p.: 93–96 °C. ^1^H-NMR (CDCl_3_) δ 6.89 (s, 2H, Ar), 4.64 (s, 1H, NH), 3.71 (s, 2H, CH_2_), 2.58 (s, 6H, (CH_3_)_2_), 2.23 (s, 3H, CH_3_), 2.02 (t, *J* = 2.5 Hz, 1H, CCH). ^13^C-NMR (CDCl_3_) δ 141.53, 138.31, 132.27 , 130.95 (Ar), 76.79, 71.68 (CCH), 31.32 (CH_2_), 21.95, 19.94 (CH_3_). HRMS (ESI) calcd. for C_12_H_16_NO_2_S [M + H]^+^: 238.0906, found: 238.0902. IR: 3319, 1429, 1321, 1162, 1076, 816, 691, 660, 529 cm^−1^.

*2-Chloro-N-(prop-2-yn-1-yl)benzenesulfonamide* (**9i**). White solid, yield: 67.0%, m.p.: 90–92 °C. ^1^H-NMR (CDCl_3_) δ 8.03 (s, 1H, Ar), 7.40 (d, *J* = 36.7 Hz, 3H, Ar), 5.26 (s, 1H, NH), 3.79 (s, 2H, CH_2_), 1.90 (s, 1H, CCH). ^13^C-NMR (CDCl_3_) δ 136.18, 132.93, 130.80, 130.46, 130.30, 126.17 (Ar), 76.46, 71.86 (CCH), 31.95 (CH_2_). HRMS (ESI) calcd. for C_9_H_9_ClNO_2_S [M + H]^+^: 230.0047, found: 230.0043. IR: 3277, 1455, 1428, 1330, 1168, 1074, 1043, 765, 666, 569, 553 cm^−1^.

#### 3.1.2. General Procedure for the Synthesis of Compounds **11a~l**

Alkyne intermediates **9a~i** (2 mmol), azide derivatives **10a~d** (2 mmol), CuSO_4_·5H_2_O (0.4 mmol) and sodium ascorbate (0.2 mmol) were dissolved in THF/H_2_O (8 mL/8 mL) and stirred for 10 h at room temperature. Upon completion of the reactions, the precipitated product was filtered to afford products **11a~l**, which were purified with column chromatography on silica gel (hexane/EtOAc = 8/1). Copies of ^1^H- and ^13^C-NMR spectras for compounds **9a~i** were shown in Supplementary materials.

*N-((1-(3-Phenoxybenzyl)-1H-1,2,3-triazol-4-yl)methyl)thiophene-2-sulfonamide* (**11a**). White solid, yield: 60.1%, m.p.: 104–106 °C. ^1^H-NMR (DMSO-*d*_6_) δ 8.34 (s, 1H, NH), 7.95 (s, 1H, Ar), 7.88 (dd, *J* = 5.0, 1.3 Hz, 1H, Ar), 7.57 (dd, *J* = 3.7, 1.3 Hz, 1H, Ar), 7.40 (tt, *J* = 9.4, 5.1 Hz, 3H, Ar), 7.15 (ddd, *J* = 11.0, 6.2, 2.3 Hz, 2H, Ar), 7.08–6.97 (m, 4H, Ar), 6.94 (dd, *J* = 8.1, 1.8 Hz, 1H, Ar), 5.54 (s, 2H, PhCH_2_), 4.12 (s, 2H, NH*CH*_2_). ^13^C-NMR (DMSO-*d*_6_) δ 157.38, 156.69, 144.03, 141.65, 138.60, 132.96, 132.18, 130.90, 130.61, 128.10, 124.21, 123.98, 123.34, 119.27, 118.53, 118.43 (Ar), 52.77 (PhCH_2_), 38.75 (NH*CH*_2_). HRMS (ESI) calcd. for C_20_H_19_N_4_O_3_S_2_ [M + H]^+^: 427.0898, found: 427.0899. IR: 3176, 3100, 1489, 1331, 1253, 1215, 1151, 750, 688, 593, 529 cm^−1^.

*5-Chloro-N-((1-(3-phenoxybenzyl)-1H-1,2,3-triazol-4-yl)methyl)thiophene-2-sulfonamide* (**11b**). White solid, yield: 68.3%, m.p.: 110–111 °C. ^1^H-NMR (DMSO-*d*_6_) δ 8.52 (s, 1H, NH), 8.00 (s, 1H, Ar), 7.55–7.29 (m, 4H, Ar), 7.25–7.10 (m, 2H, Ar), 7.07–6.97 (m, 4H, Ar), 6.98–6.88 (m, 1H, Ar), 5.55 (s, 2H, PhCH_2_), 4.15 (s, 2H, NH*CH*_2_). ^13^C-NMR (DMSO-*d*_6_) δ 157.39, 156.68, 143.77, 140.18, 138.60, 134.89, 132.04, 130.91, 130.61, 128.30, 124.21, 124.09, 123.28, 119.27, 118.52, 118.43 (Ar), 52.78 (PhCH_2_), 38.66 (NH*CH*_2_). HRMS (ESI) calcd. for C_20_H_18_ClN_4_O_3_S_2_ [M + H]^+^: 461.0512, found: 461.0509. IR: 3120, 1488, 1412, 1328, 1252, 1159, 786, 685, 612, 523 cm^−1^.

*5-Bromo-N-((1-(3-phenoxybenzyl)-1H-1,2,3-triazol-4-yl)methyl)thiophene-2-sulfonamide* (**11c**). White solid, yield: 59.1%, m.p.: 111–112 °C. ^1^H-NMR (DMSO-*d*_6_) δ 8.50 (s, 1H, NH), 8.00 (s, 1H, Ar), 7.48–7.33 (m, 4H, Ar), 7.29 (d, *J* = 4.0 Hz, 1H, Ar), 7.16 (t, *J* = 7.4 Hz, 1H, Ar), 7.08–6.98 (m, 4H, Ar), 6.94 (dd, *J* = 8.1, 1.8 Hz, 1H, Ar), 5.55 (s, 2H, PhCH_2_), 4.14 (s, 2H, NH*CH*_2_). ^13^C-NMR (DMSO-*d*_6_) δ 157.39, 156.68, 143.79, 142.76, 138.60, 132.79, 131.69, 130.91, 130.61, 124.21, 124.09, 123.28, 119.28, 118.70, 118.52, 118.43 (Ar), 52.78 (PhCH_2_), 38.67 (NH*CH*_2_). HRMS (ESI) calcd. for C_20_H_18_BrN_4_O_3_S_2_ [M + H]^+^: 505.0007, found: 505.0004. IR: 3118, 1489, 1403, 1328, 1253, 1158, 1086, 819, 786, 749, 605, 522 cm^−1^.

*2-Oxo-N-((1-(3-phenoxybenzyl)-1H-1,2,3-triazol-4-yl)methyl)-2H-chromene-6-sulfonamide* (**11d**). White solid, yield: 61.9%, m.p.: 144–146 °C. ^1^H-NMR (DMSO-*d*_6_) δ 8.28 (s, 1H, NH), 8.19 (dd, *J* = 9.1, 5.9 Hz, 2H, Ar), 8.00–7.87 (m, 2H, Ar), 7.53 (d, *J* = 8.7 Hz, 1H, Ar), 7.38 (dt, *J* = 16.3, 7.9 Hz, 3H, Ar), 7.16 (t, *J* = 7.4 Hz, 1H, Ar), 7.06–6.96 (m, 4H, Ar), 6.93 (d, *J* = 8.2 Hz, 1H, Ar), 6.63 (d, *J* = 9.6 Hz, 1H, Ar), 5.51 (s, 2H, PhCH_2_), 4.10 (s, 2H, NH*CH*_2_). ^13^C-NMR (DMSO-*d*_6_) δ 159.75, 157.37, 156.67, 156.01, 144.08, 144.01, 138.57, 136.90, 130.85, 130.60, 130.12, 127.96, 124.21, 124.00, 123.19, 119.27, 119.25, 118.46, 118.40, 118.07, 117.93 (Ar), 52.72 (PhCH_2_), 38.55 (NH*CH*_2_). HRMS (ESI) calcd. for C_25_H_21_N_4_O_5_S [M + H]^+^: 489.1237, found: 489.1233. IR: 3256, 3137, 1744, 1488, 1321, 1252, 1157, 1107, 834, 751, 601 cm^−1^.

*N-((1-(3-Phenoxybenzyl)-1H-1,2,3-triazol-4-yl)methyl)benzenesulfonamide* (**11e**). White solid, yield: 56.8%, m.p.: 127–130 °C. ^1^H-NMR (DMSO-*d*_6_) δ 8.14 (s, 1H, NH), 7.89 (s, 1H, Ar), 7.77 (dd, *J* = 5.3, 3.4 Hz, 2H, Ar), 7.69–7.47 (m, 3H, Ar), 7.47–7.27 (m, 3H, Ar), 7.16 (t, *J* = 7.4 Hz, 1H, Ar), 7.09–6.84 (m, 5H, Ar), 5.51 (s, 2H, PhCH_2_), 4.05 (s, 2H, NH*CH*_2_). ^13^C-NMR (DMSO-*d*_6_) δ 157.37, 156.69, 144.17, 140.82, 138.58, 132.86, 130.89, 130.61, 129.57, 126.97, 124.21, 123.92, 123.32, 119.26, 118.52, 118.42 (Ar), 52.74 (PhCH_2_), 38.55 (NH*CH*_2_). HRMS (ESI) calcd. for C_22_H_21_N_4_O_3_S [M + H]^+^: 421.1338, found: 421.1334. IR: 3269, 3123, 1587, 1495, 1322, 1253, 1215, 1157, 690, 587 cm^−1^.

*4-Methyl-N-((1-(3-phenoxybenzyl)-1H-1,2,3-triazol-4-yl)methyl)benzenesulfonamide* (**11f**). White solid, yield: 66.5%, m.p.: 118–121 °C. ^1^H-NMR (DMSO-*d*_6_) δ 8.03 (s, 1H, NH), 7.91 (s, 1H, Ar), 7.66 (d, *J* = 8.2 Hz, 2H, Ar), 7.52–7.27 (m, 5H, Ar), 7.16 (t, *J* = 7.4 Hz, 1H, Ar), 7.08–6.97 (m, 4H, Ar), 6.94 (dd, *J* = 8.0, 2.1 Hz, 1H, Ar), 5.52 (s, 2H, PhCH_2_), 4.01 (s, 2H, NH*CH*_2_), 2.37 (s, 3H, CH_3_). ^13^C-NMR (DMSO-*d*_6_) δ 156.88, 156.20, 143.76, 142.62, 138.11, 137.45, 130.37, 130.10, 129.51, 126.56, 123.70, 123.42, 122.78, 118.76, 118.01, 117.92 (Ar), 52.25 (PhCH_2_), 38.07 (NH*CH*_2_), 20.93 (CH_3_). HRMS (ESI) calcd. for C_23_H_23_N_4_O_3_S [M + H]^+^: 435.1497, found: 435.1491. IR: 3294, 3251, 1590, 1491, 1328, 1265, 1160, 819, 755, 687, 557 cm^−1^.

*4-(Tert-butyl)-N-((1-(3-phenoxybenzyl)-1H-1,2,3-triazol-4-yl)methyl)benzenesulfonamide* (**11g**). White solid, yield: 49.3%, m.p.: 137–139 ^°^C. ^1^H-NMR (DMSO-*d*_6_) δ 8.05 (s, 1H, NH), 7.95 (s, 1H, Ar), 7.72 (d, *J* = 8.5 Hz, 2H, Ar), 7.59 (d, *J* = 8.6 Hz, 2H, Ar), 7.39 (dt, *J* = 11.7, 8.1 Hz, 3H, Ar), 7.16 (t, *J* = 7.4 Hz, 1H, Ar), 7.10–6.84 (m, 5H, Ar), 5.53 (s, 2H, PhCH_2_), 4.02 (s, 2H, NH*CH*_2_), 1.30 (s, 9H, CH_3_). ^13^C-NMR (DMSO-*d*_6_) δ 157.39, 156.68, 155.88, 144.32, 138.64, 137.88, 130.89, 130.60, 126.94, 126.46, 124.21, 123.98, 123.26, 119.27, 118.46, 118.41 (Ar), 52.76 (PhCH_2_), 38.61 (NH*CH*_2_), 35.30 (*C*(CH_3_)_3_), 31.28 (C(*C*H_3_)_3_). HRMS (ESI) calcd. for C_26_H_29_N_4_O_3_S [M + H]^+^: 477.1967, found: 477.1960. IR: 3268, 1593, 1488, 1322, 1242, 1163, 1047, 754, 570 cm^−1^.

*2,4,6-Trimethyl-N-((1-(3-phenoxybenzyl)-1H-1,2,3-triazol-4-yl)methyl)benzenesulfonamide* (**11h**). White solid, yield: 78.7%, m.p.: 111–113 °C. ^1^H-NMR (DMSO-*d*_6_) δ 7.95 (s, 1H, NH), 7.70 (s, 1H, Ar), 7.48–7.28 (m, 3H, Ar), 7.17 (dd, *J* = 10.6, 4.2 Hz, 1H, Ar), 7.06–6.89 (m, 7H, Ar), 5.48 (s, 2H, PhCH_2_), 4.04 (s, 2H, NH*CH*_2_), 2.50 (s, 6H, CH_3_), 2.22 (s, 3H, CH_3_). ^13^C-NMR (DMSO-*d*_6_) δ 157.34, 156.71, 144.46, 141.83, 138.74, 138.60, 134.94, 132.01, 130.87, 130.61, 124.20, 123.61, 123.26, 119.22, 118.47, 118.43 (Ar), 52.65 (PhCH_2_), 37.67 (NH*CH*_2_), 22.98 (CH_3_), 20.84(CH_3_). HRMS (ESI) calcd. for C_25_H_27_N_4_O_3_S [M + H]^+^: 463.1807, found: 463.1804. IR: 3307, 3269, 1587, 1488, 1326, 1257, 1243, 1154, 736, 657 cm^−1^.

*2-Chloro-N-((1-(3-phenoxybenzyl)-1H-1,2,3-triazol-4-yl)methyl) benzenesulfonamide* (**11i**). White solid, yield: 80.8%, m.p.: 97–99 °C. ^1^H-NMR (DMSO-*d*_6_) δ 8.41 (s, 1H, NH), 7.91 (dd, *J* = 7.8, 1.5 Hz, 1H, Ar), 7.83 (s, 1H, Ar), 7.61–7.48 (m, 2H, Ar), 7.48–7.32 (m, 4H, Ar), 7.17 (t, *J* = 7.4 Hz, 1H, Ar), 7.10–6.89 (m, 5H, Ar), 5.50 (s, 2H, PhCH_2_), 4.17 (s, 2H, NH*CH*_2_). ^13^C-NMR (DMSO-*d*_6_) δ 157.36, 156.70, 144.14, 138.53, 138.49, 134.27, 131.99, 131.05, 130.89, 130.79, 130.62, 127.91, 124.21, 123.83, 123.34, 119.26, 118.54, 118.44 (Ar), 52.67 (PhCH_2_), 38.31 (NH*CH*_2_). HRMS (ESI) calcd. for C_22_H_20_ClN_4_O_3_S [M + H]^+^: 455.0948, found: 455.0945. IR: 3137, 1593, 1489, 1451, 1332, 1253, 1212, 1157, 758, 688, 585 cm^−1^.

*N-((1-(3-Cyanobenzyl)-1H-1,2,3-triazol-4-yl)methyl)-4-methylbenzenesulfonamide* (**11j**). White solid, yield: 58.1%, m.p.: 145–147 °C. ^1^H-NMR (DMSO-*d*_6_) δ 8.03 (s, 1H, NH), 7.98 (s, 1H, Ar), 7.83 (td, *J* = 4.6, 1.4 Hz, 1H, Ar), 7.77 (s, 1H, Ar), 7.66 (d, *J* = 8.2 Hz, 2H, Ar), 7.60 (d, *J* = 5.0 Hz, 2H, Ar), 7.34 (d, *J* = 8.0 Hz, 2H, Ar), 5.61 (s, 2H, PhCH_2_), 4.02 (s, 2H, NH*CH*_2_), 2.37 (s, 3H, CH_3_). ^13^C-NMR (DMSO-*d*_6_) δ 143.89, 142.62, 137.55, 137.44, 132.88, 131.95, 131.56, 130.03, 129.50, 126.56, 123.62, 118.40, 111.64 (Ar), 51.70 (PhCH_2_), 38.05 (NH*CH*_2_), 20.93 (CH_3_). HRMS (ESI) calcd. for C_18_H_18_N_5_O_2_S [M + H]^+^: 368.1181, found: 368.1181. IR: 3265, 2217, 1430, 1323, 1160, 1049, 889, 709, 546 cm^−1^.

*4-Methyl-N-((1-(3-methylbenzyl)-1H-1,2,3-triazol-4-yl)methyl)benzenesulfonamide* (**11k**). White solid, yield: 67.7%, m.p.: 145–148 °C. ^1^H-NMR (DMSO-*d*_6_) δ 8.01 (t, *J* = 6.0 Hz, 1H, NH), 7.87 (s, 1H, Ar), 7.66 (d, *J* = 8.1 Hz, 2H, Ar), 7.34 (d, *J* = 8.0 Hz, 2H, Ar), 7.25 (t, *J* = 7.5 Hz, 1H, Ar), 7.14 (dd, *J* = 15.2, 7.5 Hz, 2H, Ar), 7.06 (d, *J* = 7.6 Hz, 1H, Ar), 5.47 (s, 2H, PhCH_2_), 4.00 (d, *J* = 6.0 Hz, 2H, NH*CH*_2_), 2.37 (s, 3H, CH_3_), 2.29 (s, 3H, CH_3_). ^13^C-NMR (DMSO-*d*_6_) δ 143.70, 142.61, 137.92, 137.44, 135.83, 129.51, 128.73, 128.61, 128.55, 126.57, 125.06, 123.27 (Ar), 52.69 (PhCH_2_), 38.09 (NH*CH*_2_), 20.94 (CH_3_), 20.89 (CH_3_). HRMS (ESI) calcd. for C_18_H_21_N_4_O_2_S [M + H]^+^: 357.1388, found: 357.1385. IR: 3258, 3121, 1452, 1325, 1160, 1098, 772, 669, 558 cm^−1^.

*N-((1-(3-Methoxybenzyl)-1H-1,2,3-triazol-4-yl)methyl)-4-methylbenzenesulfonamide* (**11l**). White solid, yield: 82.8%, m.p.: 112–114 °C. ^1^H-NMR (DMSO-*d*_6_) δ 8.02 (s, 1H, NH), 7.89 (s, 1H, Ar), 7.66 (d, *J* = 8.2 Hz, 2H, Ar), 7.34 (d, *J* = 8.1 Hz, 2H, Ar), 7.28 (t, *J* = 7.9 Hz, 1H, Ar), 6.99–6.85 (m, 2H, Ar), 6.82 (d, *J* = 7.6 Hz, 1H, Ar), 5.49 (s, 2H, PhCH_2_), 4.01 (s, 2H, NH*CH*_2_), 3.74 (s, 3H, OCH_3_), 2.37 (s, 3H, CH_3_). ^13^C-NMR (DMSO-*d*_6_) δ 159.40, 143.72, 142.62, 137.45, 137.38, 129.84, 129.51, 126.56, 123.34, 120.00, 113.79, 113.37 (Ar), 55.08 (OCH_3_), 52.60 (PhCH_2_), 38.08 (NH*CH*_2_), 20.92 (CH_3_). HRMS (ESI) calcd. for C_18_H_21_N_4_O_3_S [M + H]^+^: 373.1339, found: 373.1334. IR: 3294, 3247, 1600, 1322, 1286, 1265, 1091, 1040, 775, 668, 557 cm^−1^.

### 3.2. MTT Assay

MGC-803 cells (human gastric cancer), MCF-7 (human breast cancer), PC-3 (human prostate cancer) and GES-1 (human gastric epithelial cell) were cultured in RPMI 1640 medium with 10% FBS and 100 U/mL penicillin and 0.1 mg/mL streptomycin in the 37 °C in an atmosphere containing 5% CO_2_. All cell lines were purchased from the China Center for Type Culture Collection (CCTCC, Shanghai, China). For pharmacological investigations, 10 mM stock solutions of the tested compounds were prepared with dimethyl sulfoxide (DMSO). The highest DMSO concentration of the medium (0.1%) did not have any substantial effect on the determined cellular functions. The MTT assay was prepared according to the previous method [26,27,28].

### 3.3. In Vitro Tubulin Polymerization Inhibition Assay

Tubulin polymerization in vitro was assyed with 100 μL samples and monitored by absorbance at 350 nm (Filter A00019x) in 96-well plates with an Appliskan plate reader (Thermo Fisher Scientific, Waltham, MA, USA). An amount of 5.6 mg/mL tubulin was resuspended in PEM buffer (80 mM PIPES (pH 6.9), 1 mM EGTA, 0.5 mM MgCl_2_, 1 mM ATP, 10.2% (*v*/*v*) glycerol) and then was preincubated with compound **11f** or vehicle DMSO on ice. Data were exported using the Thermo Scientific SkanIt software of the Appliskan (version 2.3) and Excel software was used to generate plots. For convenience, data were normalized to the minimum absorbance value of the initial stable plateau. This tubulin polymerization inhibition assay was measured according to the method originally [6,25].

## 4. Conclusions

In summary, a series of novel sulfanilamide-1,2,3-triazole hybrids were designed, synthesized and evaluated for their antiproliferative activity against three selected cancer cell lines (MGC-803, MCF-7 and PC-3). Most of the synthesized compounds exhibited moderate to good activity against all the selected cancer cell lines. Particularly, compound **11f** showed the most excellent antiproliferative activity, with an IC_50_ value of 4.1 μM against PC-3 cancer cells. The tubulin polymerization inhibition activity of compound **11f** was 2.41 μM. Highlights of structure activity relationships were the importance of 1,2,3-triazole scaffold and phenoxy group. Efforts to optimize the structure of compound **11f** to further improve its potency are ongoing.

Although **CA-4P** targeting the colchicine binding site was designated with orphan drug status for the treatment of anaplastic thyroid cancer and ovarian cancer by the FDA [29], two major problems are still encountered during its use as therapeutics: (1) the isomerization to the inactive *trans* configuration during storage by heat and light [30]; (2) the undesirable side effects and potent toxicity against normal cells [31]. Compared to **CA-4P**, there is no isomerized double bond in compound **11f**. Importantly, compound **11f** exhibited no significant cytotoxicity against GES-1 (>64 μM), suggesting that sulfanilamide-1,2,3-triazole hybrid **11f** could be a potent tubulin polymerization inhibitor for potential clinical application.

## Figures and Tables

**Table 1:** Antiproliferative results of the target compounds.

Compound	IC_50_ (μM) ^a^		
	MGC-803	MCF-7	PC-3
**9a**	>100	>100	>100
**9d**	>100	>100	>100
**9f**	>100	>100	>100
**11a**	17.0 ± 1.8	78.5 ± 2.2	24.6 ± 0.9
**11b**	15.3 ± 0.8	49.9 ± 1.2	19.8 ± 0.6
**11c**	31.0 ± 2.2	34.7 ± 2.6	49.4 ± 1.0
**11d**	88.8 ± 0.5	36.4 ± 2.0	34.1 ± 0.3
**11e**	21.7 ± 0.1	39.6 ± 5.0	6.4 ± 1.2
**11f**	13.7 ± 2.9	15.7 ± 0.2	4.1 ± 1.4
**11g**	>100	54.9 ± 1.7	7.1 ± 0.5
**11h**	18.4 ± 0.7	24.6 ± 1.6	24.7 ± 3.0
**11i**	15.3 ± 0.5	77.5 ± 0.7	22.7 ± 0.5
**11j**	41.3 ± 2.3	>100	>100
**11k**	75.8 ± 0.6	>100	>100
**11l**	77.2 ± 7.8	>100	>100
**5-Fu**	15.6 ± 0.4	21.2 ± 3.6	13.9 ± 1.5
**CA-4P**	0.027 ± 0.003	0.039 ± 0.005	nd ^b^

^a^ Inhibitory activity was assayed by exposure for 48 h to substances and expressed as concentration required to inhibit tumor cell proliferation by 50% (IC_50_). Data are presented as the means ± SDs of three independent experiments. ^b^ Not determined.

## Supplementary Materials

Supplementary materials are available online.

## Abbreviations

CA-4P	Combretastatin A-4P
CDK	Cyclin-dependent kinase
5-Fu	5-Fluorouracil
CLog *P*	Lipo-hydro partition coefficient
SAR	Structure-activity relationship
