# Supplementary material for: Design and Antiproliferative Evaluation of Novel Sulfanilamide Derivatives as Potential Tubulin Polymerization Inhibitors
Source: Molecules. 2017 Sep 5;22(9):1470. doi: 10.3390/molecules22091470 (PMC6151726; doi:10.3390/molecules22091470)
Supplement: Supplementary file 1 [file molecules-22-01470-s001.pdf]

# Design and antiproliferative evaluation of novel sulfanilamide derivatives as potential tubulin polymerization inhibitors

**Dong-Jun Fu[1a], Ji-Feng Liu[1a], Ruo-Han Zhao[1a], Jia-Huan Li[a], Sai-Yang Zhang[*b] and Yan-Bing Zhang[*a]**

[a]School of Pharmaceutical Sciences& Collaborative Innovation Center of New Drug Research and Safety Evaluation, Zhengzhou University, Zhengzhou 450001, China.
[b]School of Basic Medical Sciences, Zhengzhou University, Zhengzhou 450001, China.
[1]These authors contributed equally to this work.
[*]Correspondence: celeron16@163.com (S.-Y. Zhang), zhangyb@zzu.edu.cn (Y.-B. Zhang)

N-((1-(3-phenoxybenzyl)-1H-1,2,3-triazol-4-yl)methyl)thiophene-2-sulfonamide (11a)

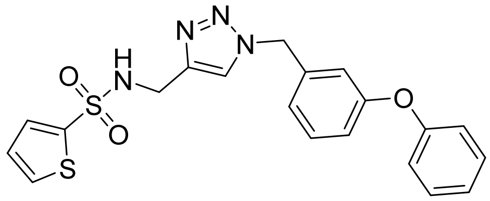


White soild, yield：60.1%, mp: 104-106°C. $^{1}$H NMR (400 MHz, DMSO) δ 8.34 (s, 1H, NH), 7.95 (s, 1H, Ar), 7.88 (dd, *J* = 5.0, 1.3 Hz, 1H, Ar), 7.57 (dd, *J* = 3.7, 1.3 Hz, 1H, Ar), 7.40 (tt, *J* = 9.4, 5.1 Hz, 3H, Ar), 7.15 (ddd, *J* = 11.0, 6.2, 2.3 Hz, 2H, Ar), 7.08 – 6.97 (m, 4H, Ar), 6.94 (dd, *J* = 8.1, 1.8 Hz, 1H, Ar), 5.54 (s, 2H, $PhCH_2$), 4.12 (s, 2H, $NHCH_2$). $^{13}$C NMR (100 MHz, DMSO) δ 157.38, 156.69, 144.03, 141.65, 138.60, 132.96, 132.18, 130.90, 130.61, 128.10, 124.21, 123.98, 123.34, 119.27, 118.53, 118.43 (Ar), 52.77 ($PhCH_2$), 38.75 ($NHCH_2$). HRMS (ESI) calcd for $C_{20}H_{19}N_4O_3S_2$ $[M+H]^+$: 427.0898, found: 427.0899. IR: 3176, 3100, 1489, 1331, 1253, 1215, 1151, 750, 688, 593, 529 $cm^{-1}$.

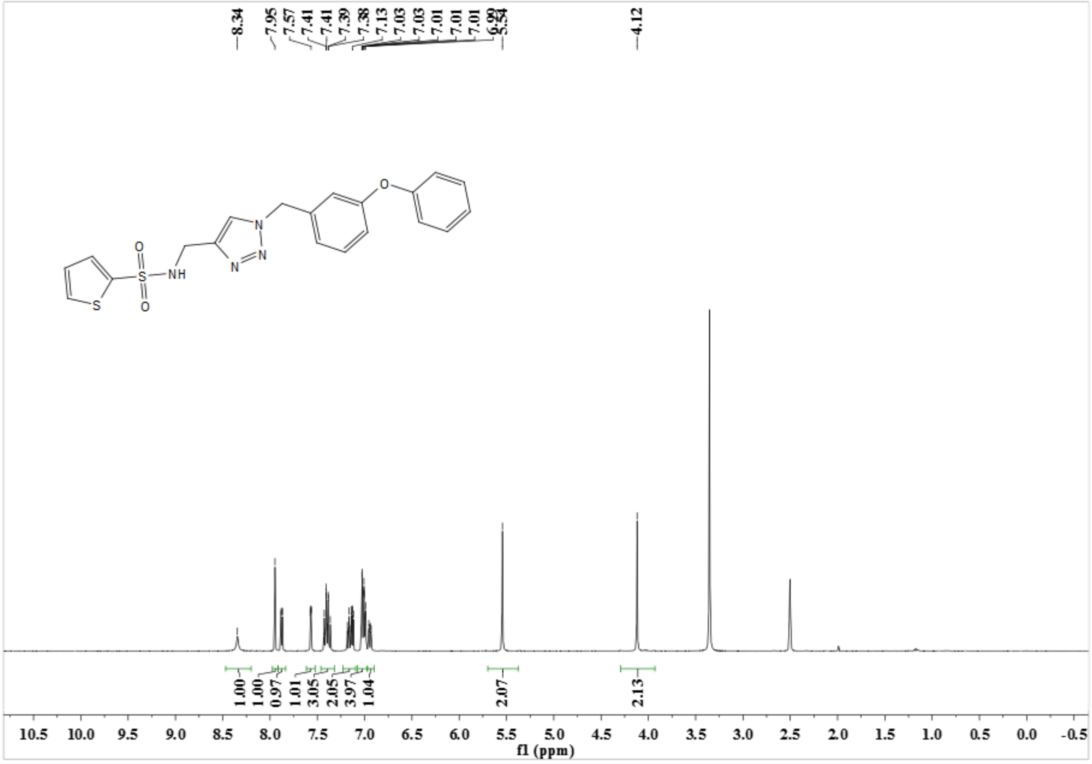


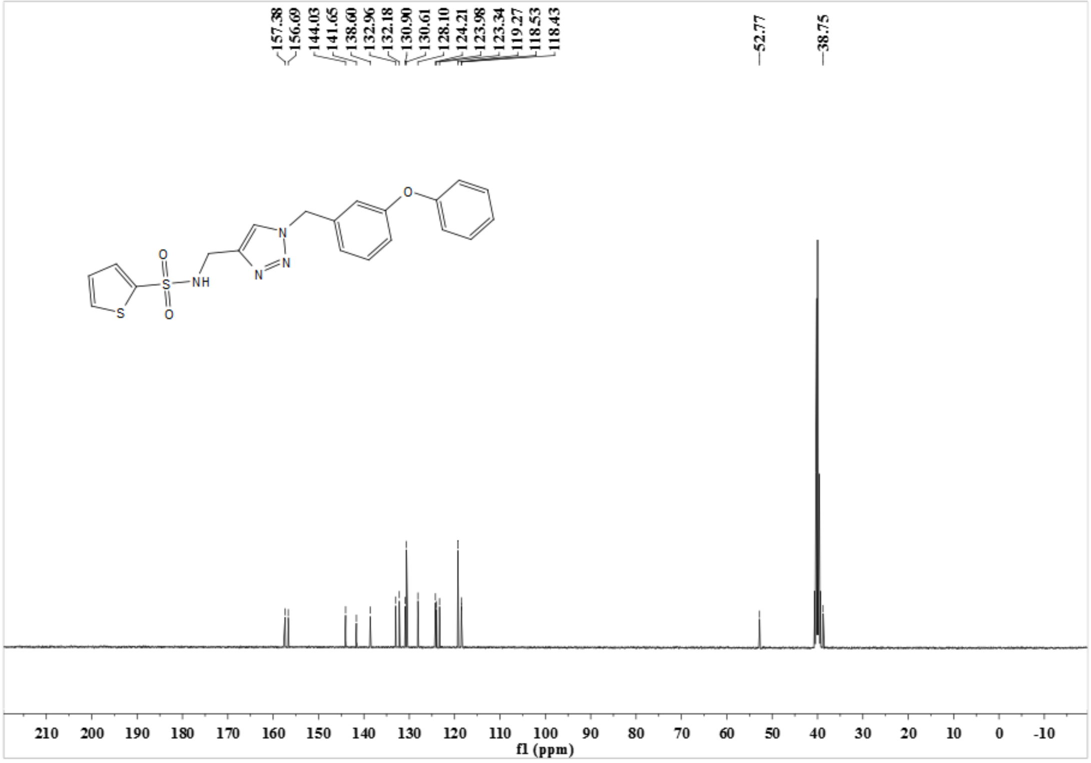


5-chloro-N-((1-(3-phenoxybenzyl)-1H-1,2,3-triazol-4-yl)methyl)thiophene-2-sulfonamide (11b)

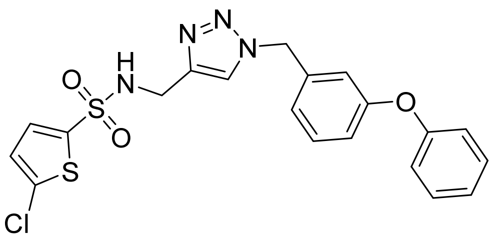

White soild, yield：68.3%, mp: 110-111ºC. $^{1}$H NMR (400 MHz, DMSO) δ 8.52 (s, 1H, NH), 8.00 (s, 1H, Ar), 7.55 – 7.29 (m, 4H, Ar), 7.25 – 7.10 (m, 2H, Ar), 7.07 – 6.97 (m, 4H, Ar), 6.98 – 6.88 (m, 1H, Ar), 5.55 (s, 2H, $PhCH_2$), 4.15 (s, 2H, NH*CH₂*). $^{13}$C NMR (100 MHz, DMSO) δ 157.39, 156.68, 143.77, 140.18, 138.60, 134.89, 132.04, 130.91, 130.61, 128.30, 124.21, 124.09, 123.28, 119.27, 118.52, 118.43 (Ar), 52.78 ($PhCH_2$), 38.66 (NH$\mathit{CH_2}$). HRMS (ESI) calcd for $C_{20}H_{18}ClN_4O_3S_2$ $[M+H]^+$: 461.0512, found: 461.0509. IR: 3120, 1488, 1412, 1328, 1252, 1159, 786, 685, 612, 523 $cm^{-1}$.

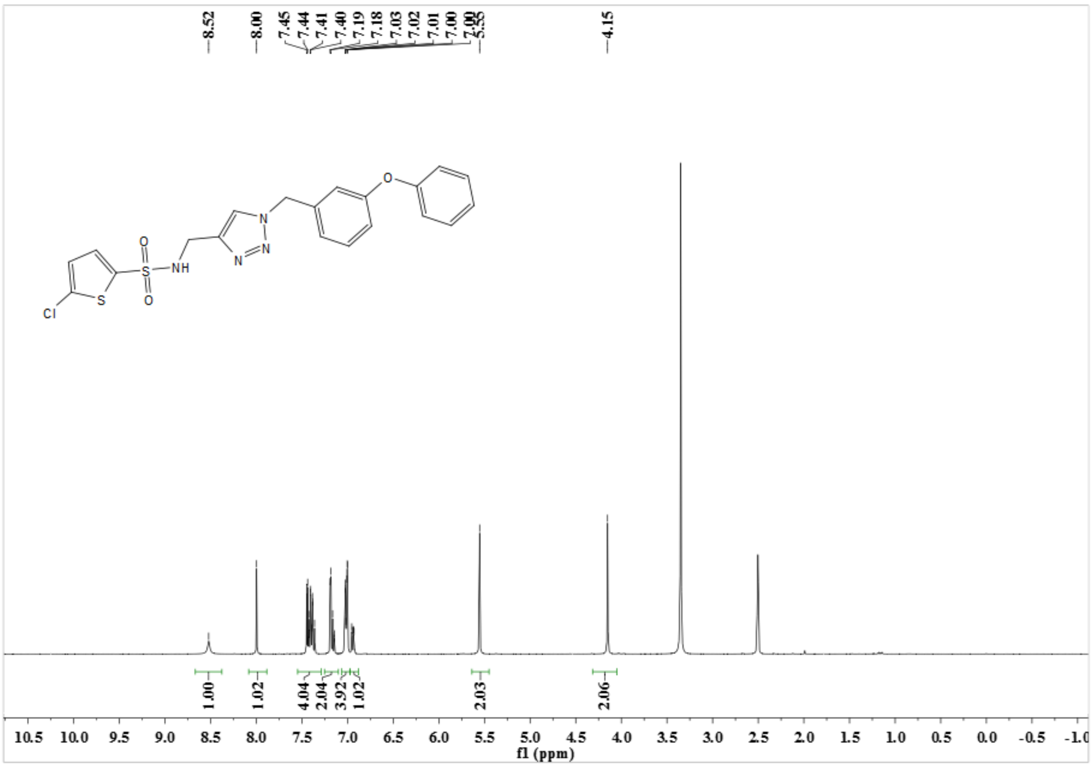

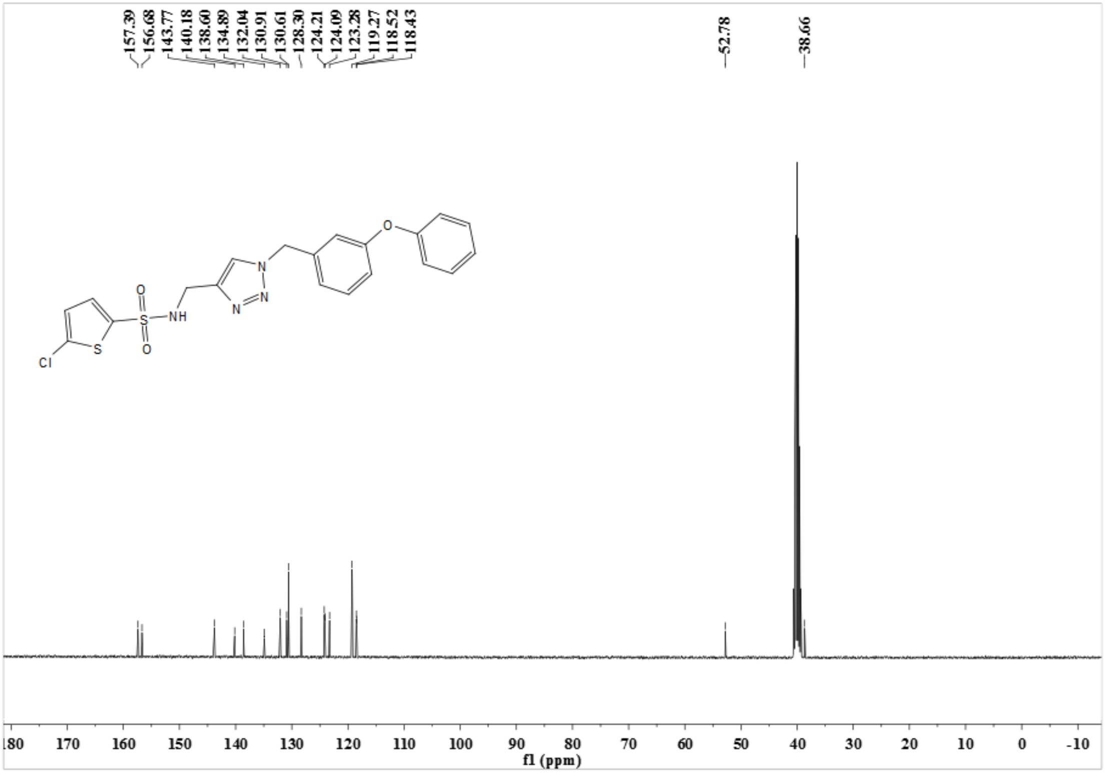


5-bromo-N-((1-(3-phenoxybenzyl)-1H-1,2,3-triazol-4-yl)methyl)thiophene-2-sulfonamide (11c)

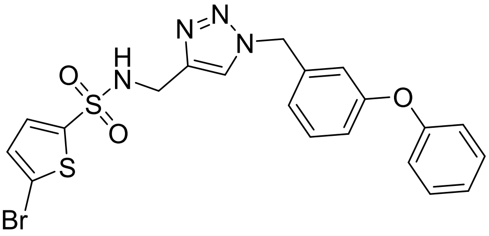

White soild, yield：59.1%, mp: 111-112°C. $^{1}$H NMR (400 MHz, DMSO) δ 8.50 (s, 1H, NH), 8.00 (s, 1H, Ar), 7.48 – 7.33 (m, 4H, Ar), 7.29 (d, *J* = 4.0 Hz, 1H, Ar), 7.16 (t, *J* = 7.4 Hz, 1H, Ar), 7.08 – 6.98 (m, 4H, Ar), 6.94 (dd, *J* = 8.1, 1.8 Hz, 1H, Ar), 5.55 (s, 2H, PhCH$_2$), 4.14 (s, 2H, NH*CH$_2$*). $^{13}$C NMR (100 MHz, DMSO) δ 157.39, 156.68, 143.79, 142.76, 138.60, 132.79, 131.69, 130.91, 130.61, 124.21, 124.09, 123.28, 119.28, 118.70, 118.52, 118.43 (Ar), 52.78 (PhCH$_2$), 38.67 (NH*CH$_2$*). HRMS (ESI) calcd for $C_{20}H_{18}BrN_4O_3S_2$ [M+H]$^+$: 505.0007, found: 505.0004. IR: 3118, 1489, 1403, 1328, 1253, 1158, 1086, 819, 786, 749, 605, 522 cm$^{-1}$.

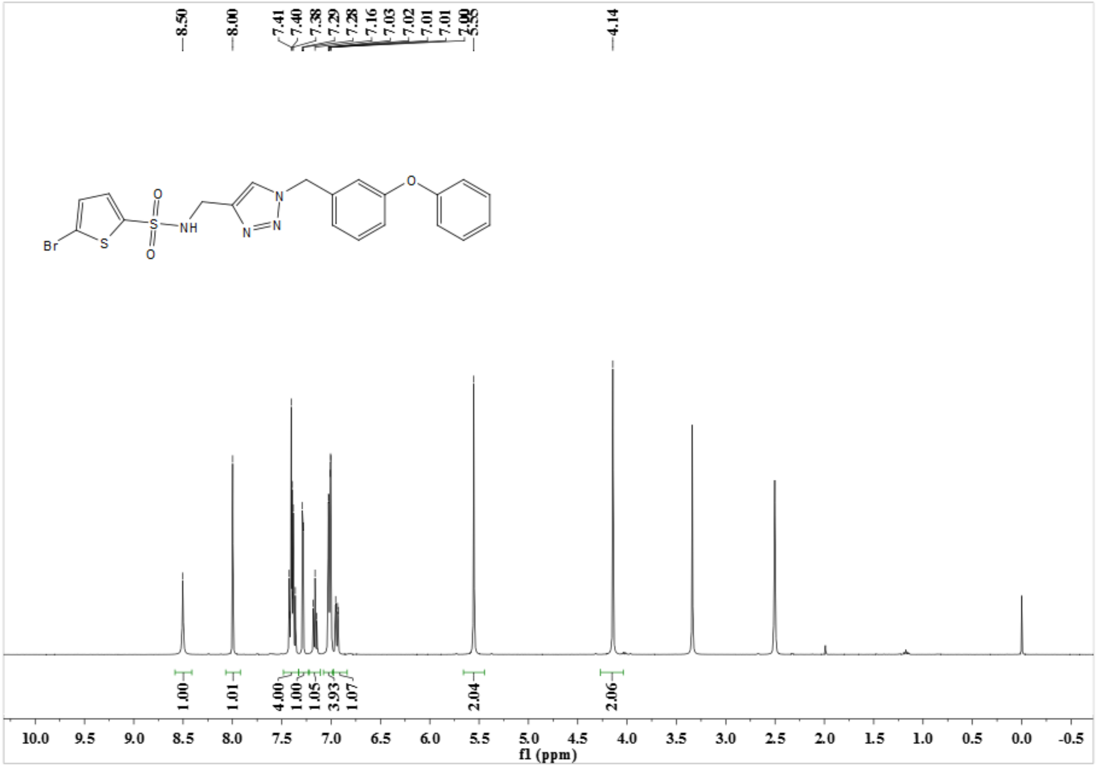


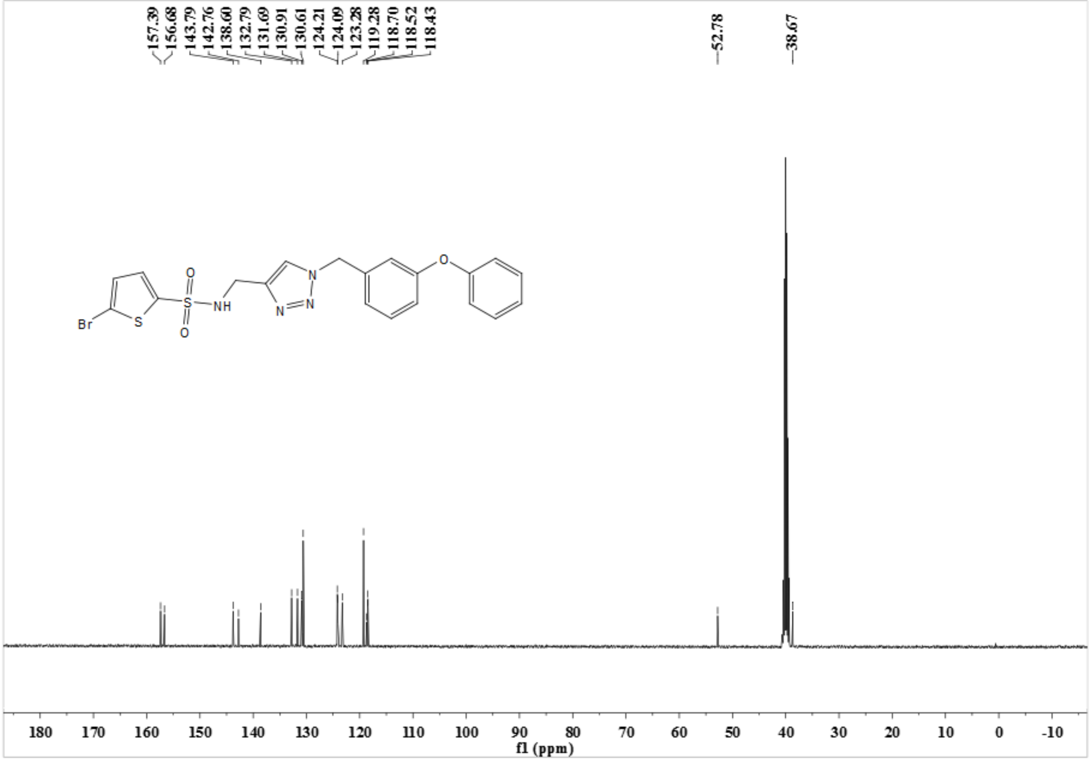


2-oxo-N-((1-(3-phenoxybenzyl)-1H-1,2,3-triazol-4-yl)methyl)-2H-chromene-6-sulfonamide (11d)

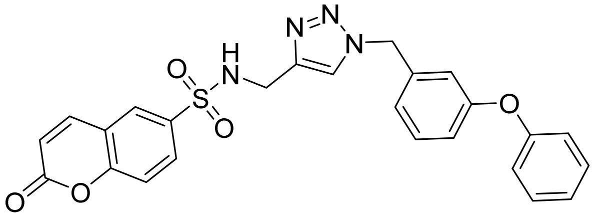

White soild, yield：61.9%, mp: 144-146°C. $^1$H NMR (400 MHz, DMSO) δ 8.28 (s, 1H, NH), 8.19 (dd, $J$ = 9.1, 5.9 Hz, 2H, Ar), 8.00 – 7.87 (m, 2H, Ar), 7.53 (d, $J$ = 8.7 Hz, 1H, Ar), 7.38 (dt, $J$ = 16.3, 7.9 Hz, 3H, Ar), 7.16 (t, $J$ = 7.4 Hz, 1H, Ar), 7.06 – 6.96 (m, 4H, Ar), 6.93 (d, $J$ = 8.2 Hz, 1H, Ar), 6.63 (d, $J$ = 9.6 Hz, 1H, Ar), 5.51 (s, 2H, $PhCH_2$), 4.10 (s, 2H, $NHCH_2$). $^{13}$C NMR (100 MHz, DMSO) δ 159.75, 157.37, 156.67, 156.01, 144.08, 144.01, 138.57, 136.90, 130.85, 130.60, 130.12, 127.96, 124.21, 124.00, 123.19, 119.27, 119.25, 118.46, 118.40, 118.07, 117.93 (Ar), 52.72 ($PhCH_2$), 38.55 ($NHCH_2$). HRMS (ESI) calcd for $C_{25}H_{21}N_4O_5S$ $[M+H]^+$: 489.1237, found: 489.1233. IR: 3256, 3137, 1744, 1488, 1321, 1252, 1157, 1107, 834, 751, 601 $cm^{-1}$.

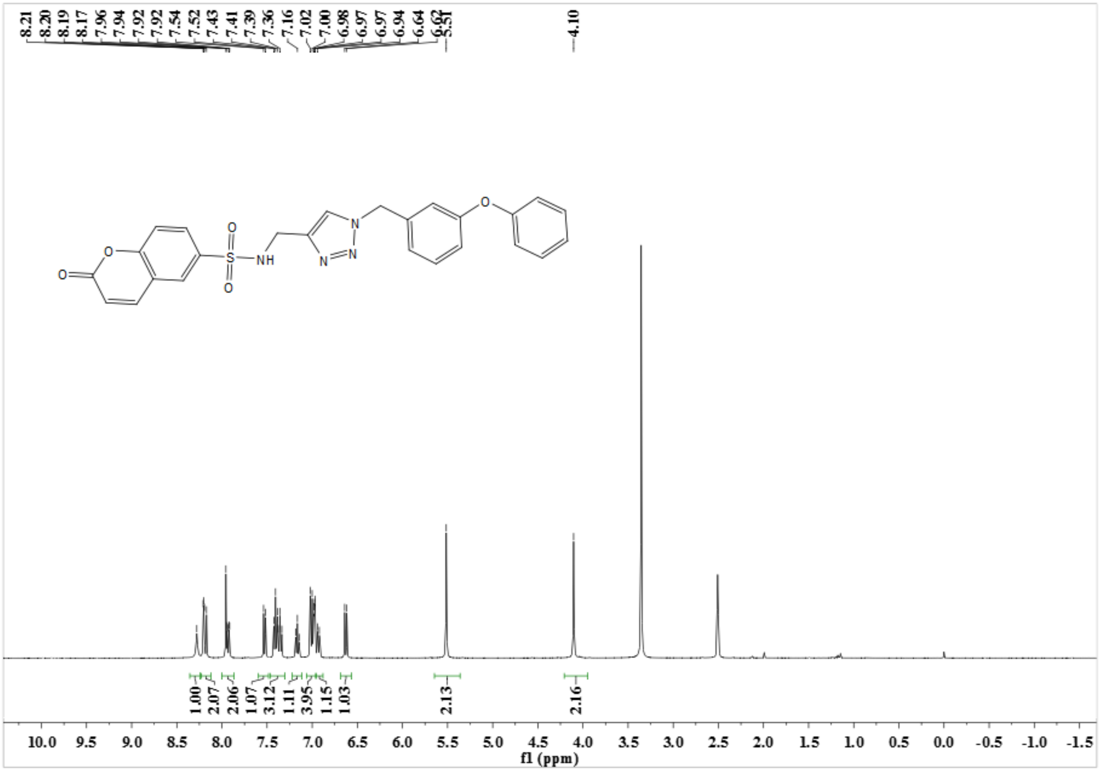

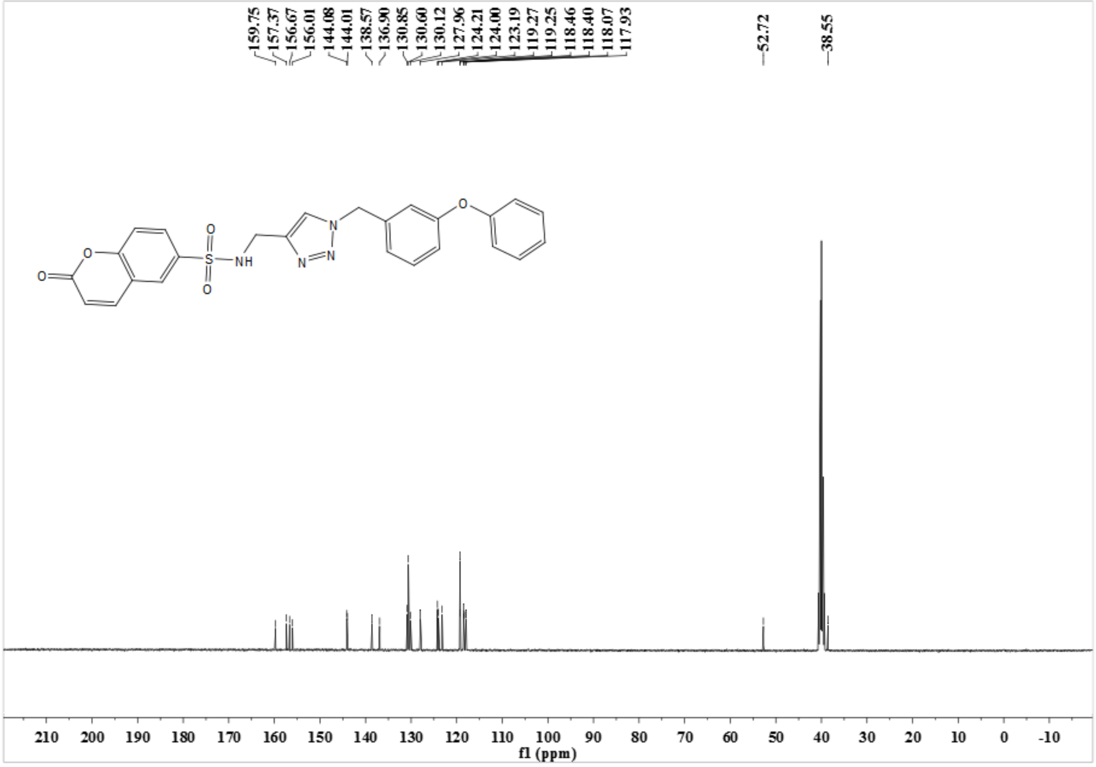


N-((1-(3-phenoxybenzyl)-1H-1,2,3-triazol-4-yl)methyl)benzenesulfonamide (11e)

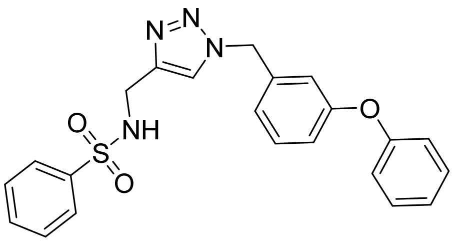

White soild, yield：56.8%, mp: 127-130$^{o}$C. $^{1}$H NMR (400 MHz, DMSO) δ 8.14 (s, 1H, NH), 7.89 (s, 1H, Ar), 7.77 (dd, $J$ = 5.3, 3.4 Hz, 2H, Ar), 7.69 – 7.47 (m, 3H, Ar), 7.47 – 7.27 (m, 3H, Ar), 7.16 (t, $J$ = 7.4 Hz, 1H, Ar), 7.09 – 6.84 (m, 5H, Ar), 5.51 (s, 2H, PhCH$_2$), 4.05 (s, 2H, NH*CH$_2$*). $^{13}$C NMR (100 MHz, DMSO) δ 157.37, 156.69, 144.17, 140.82, 138.58, 132.86, 130.89, 130.61, 129.57, 126.97, 124.21, 123.92, 123.32, 119.26, 118.52, 118.42 (Ar), 52.74 (PhCH$_2$), 38.55 (NH*CH$_2$*). HRMS (ESI) calcd for $C_{22}H_{21}N_4O_3S$ [M+H]$^+$: 421.1338, found: 421.1334. IR: 3269, 3123, 1587, 1495, 1322, 1253, 1215, 1157, 690, 587 cm$^{-1}$.

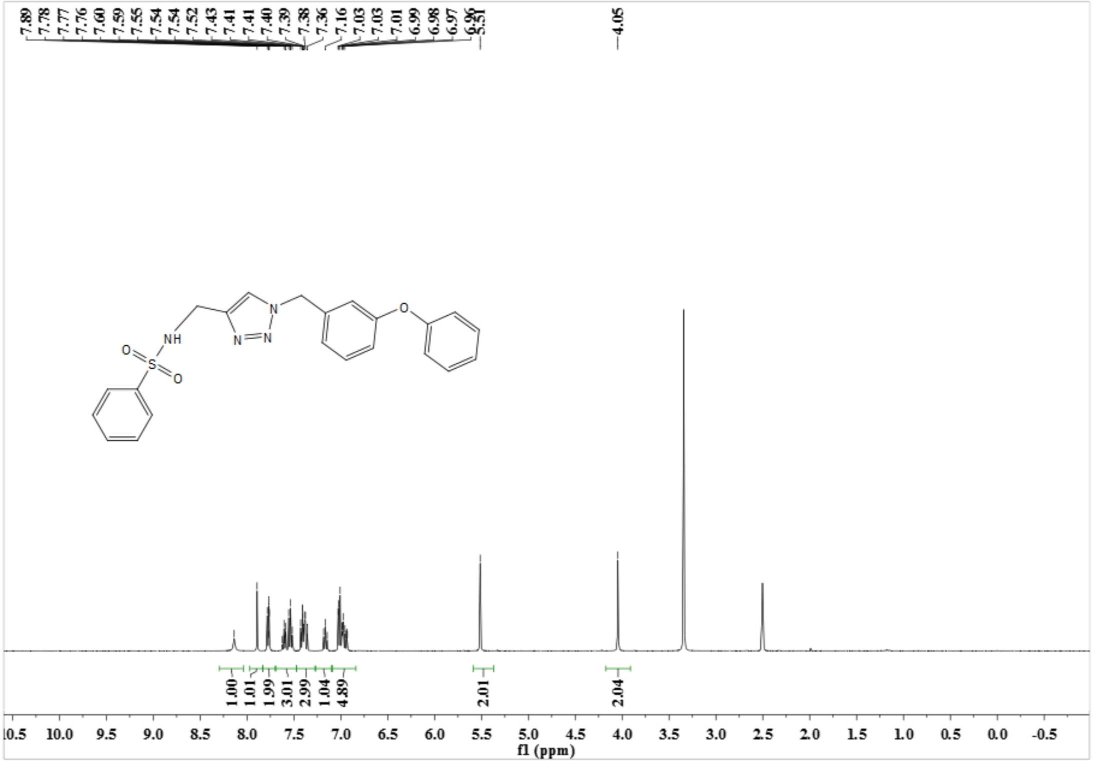

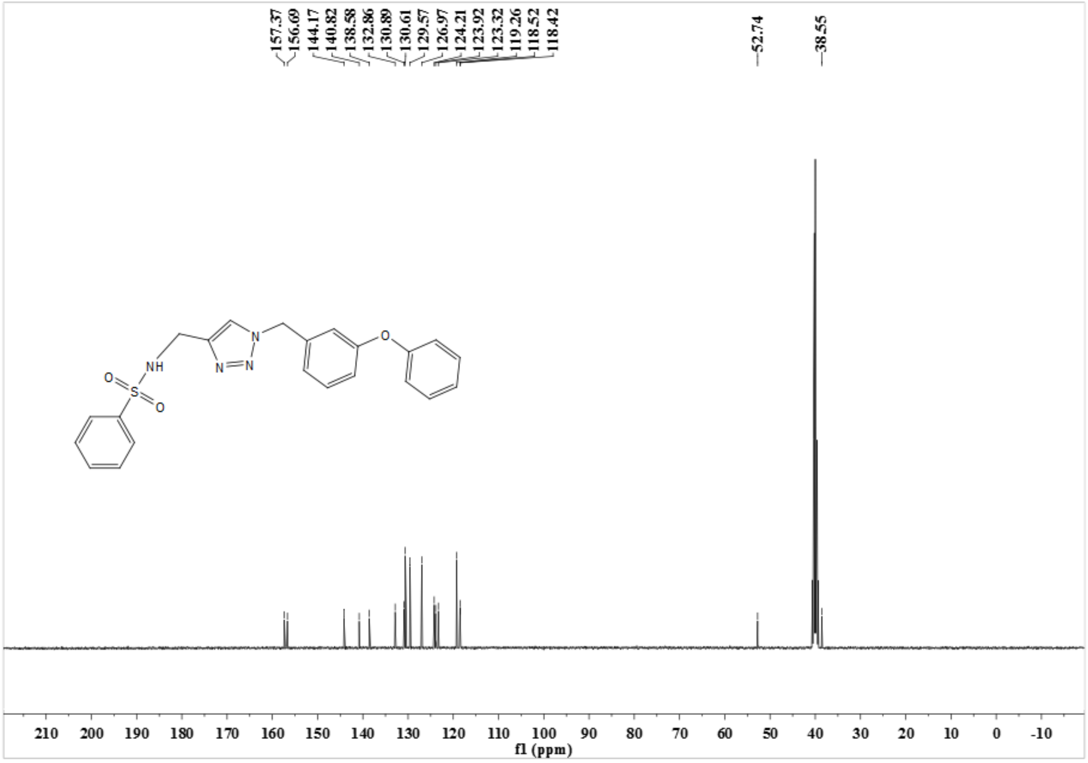

4-methyl-N-((1-(3-phenoxybenzyl)-1H-1,2,3-triazol-4-yl)methyl)benzenesulfonamide (11 f)

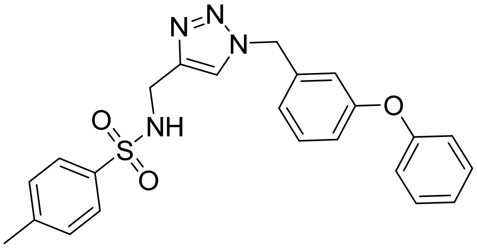

White soild, yield：66.5%, mp: 118-121°C. $^{1}$H NMR (400 MHz, DMSO) δ 8.03 (s, 1H, NH), 7.91 (s, 1H, Ar), 7.66 (d, *J* = 8.2 Hz, 2H, Ar), 7.52 – 7.27 (m, 5H, Ar), 7.16 (t, *J* = 7.4 Hz, 1H, Ar), 7.08 – 6.97 (m, 4H, Ar), 6.94 (dd, *J* = 8.0, 2.1 Hz, 1H, Ar), 5.52 (s, 2H, $PhCH_2$), 4.01 (s, 2H, NH$CH_2$), 2.37 (s, 3H, $CH_3$). $^{13}$C NMR (100MHz, DMSO) δ 156.88, 156.20, 143.76, 142.62, 138.11, 137.45, 130.37, 130.10, 129.51, 126.56, 123.70, 123.42, 122.78, 118.76, 118.01, 117.92 (Ar), 52.25 ($PhCH_2$), 38.07 (NH$CH_2$), 20.93 ($CH_3$). HRMS (ESI) calcd for $C_{23}H_{23}N_4O_3S$ $[M+H]^+$: 435.1497, found: 435.1491. IR: 3294, 3251, 1590, 1491, 1328, 1265, 1160, 819, 755, 687, 557 $cm^{-1}$.

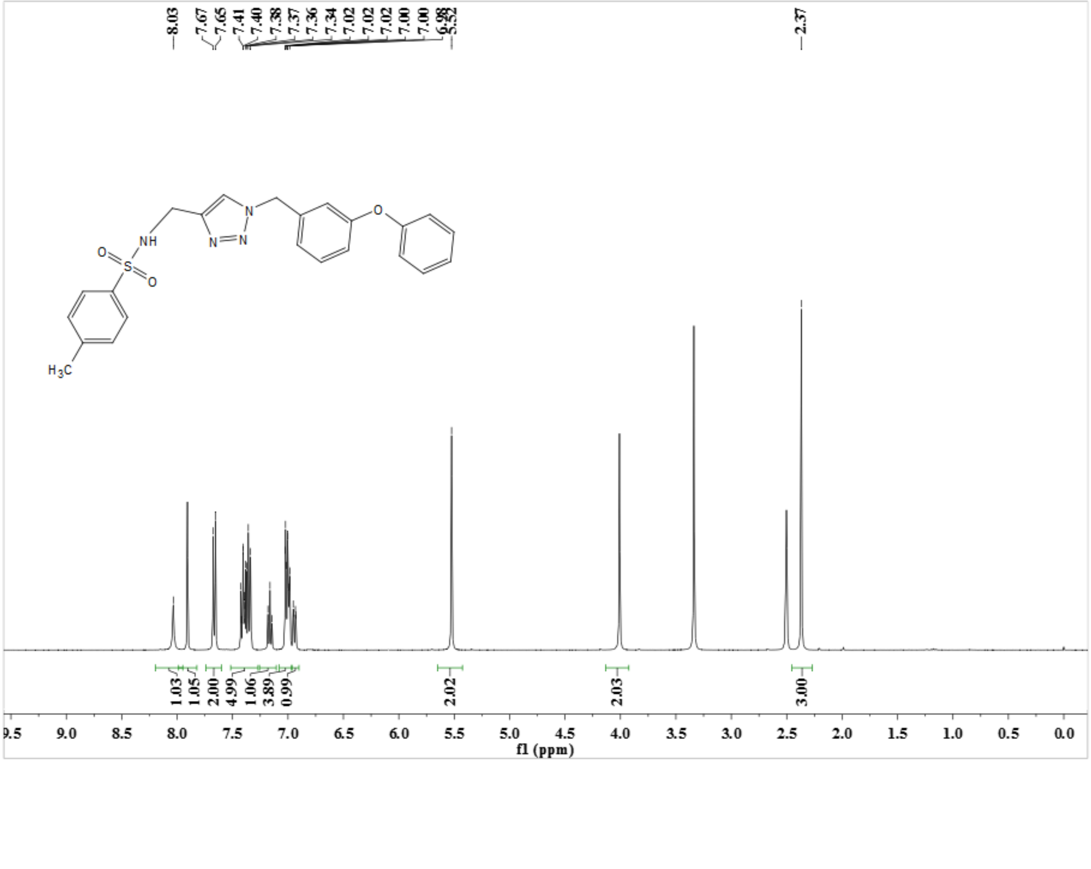

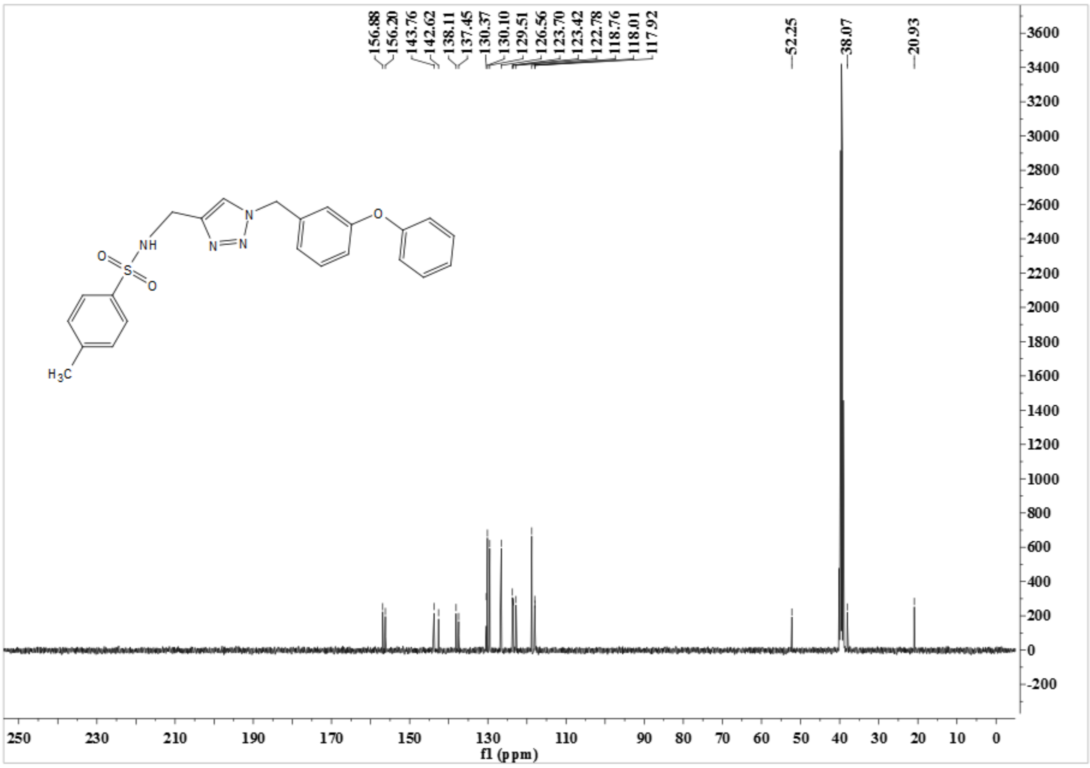

4-(tert-butyl)-N-((1-(3-phenoxybenzyl)-1H-1,2,3-triazol-4- yl)methyl) benzenesulfonamide (11g)

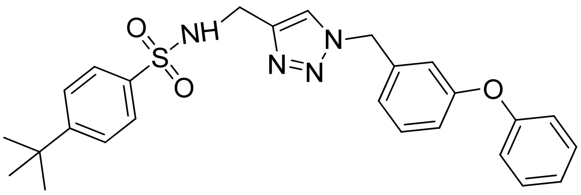

White soild, yield：49.3%, mp: 137-139°C. $^{1}$H NMR (400 MHz, DMSO) δ 8.05 (s, 1H, NH), 7.95 (s, 1H, Ar), 7.72 (d, $J$ = 8.5 Hz, 2H, Ar), 7.59 (d, $J$ = 8.6 Hz, 2H, Ar), 7.39 (dt, $J$ = 11.7, 8.1 Hz, 3H, Ar), 7.16 (t, $J$ = 7.4 Hz, 1H, Ar), 7.10 – 6.84 (m, 5H, Ar), 5.53 (s, 2H, PhCH$_2$), 4.02 (s, 2H, NH*CH$_2$*), 1.30 (s, 9H, CH$_3$). $^{13}$C NMR (100 MHz, DMSO) δ 157.39, 156.68, 155.88, 144.32, 138.64, 137.88, 130.89, 130.60, 126.94, 126.46, 124.21, 123.98, 123.26, 119.27, 118.46, 118.41 (Ar), 52.76 (PhCH$_2$), 38.61 (NH*CH$_2$*), 35.30 (*C*(CH$_3$)$_3$), 31.28 (C(*C*H$_3$)$_3$). HRMS (ESI) calcd for $C_{26}H_{29}N_4O_3S$ [M+H]$^+$: 477.1967, found: 477.1960. IR: 3268, 1593, 1488, 1322, 1242, 1163, 1047, 754, 570 cm$^{-1}$.

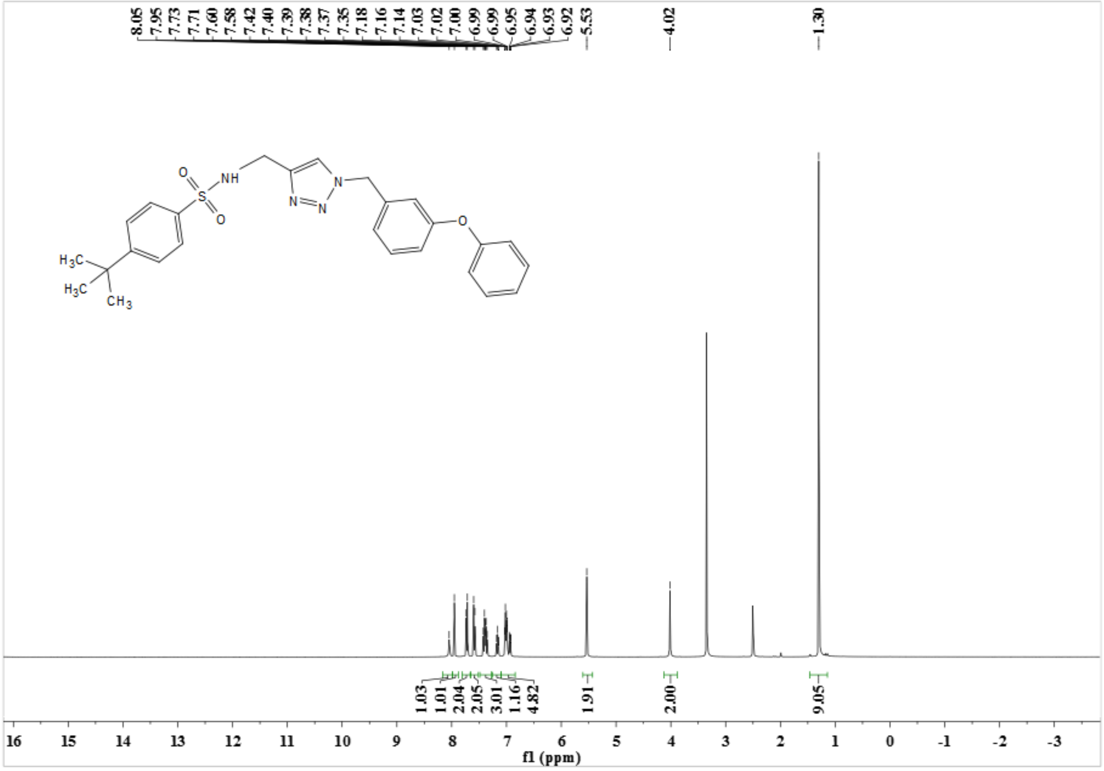


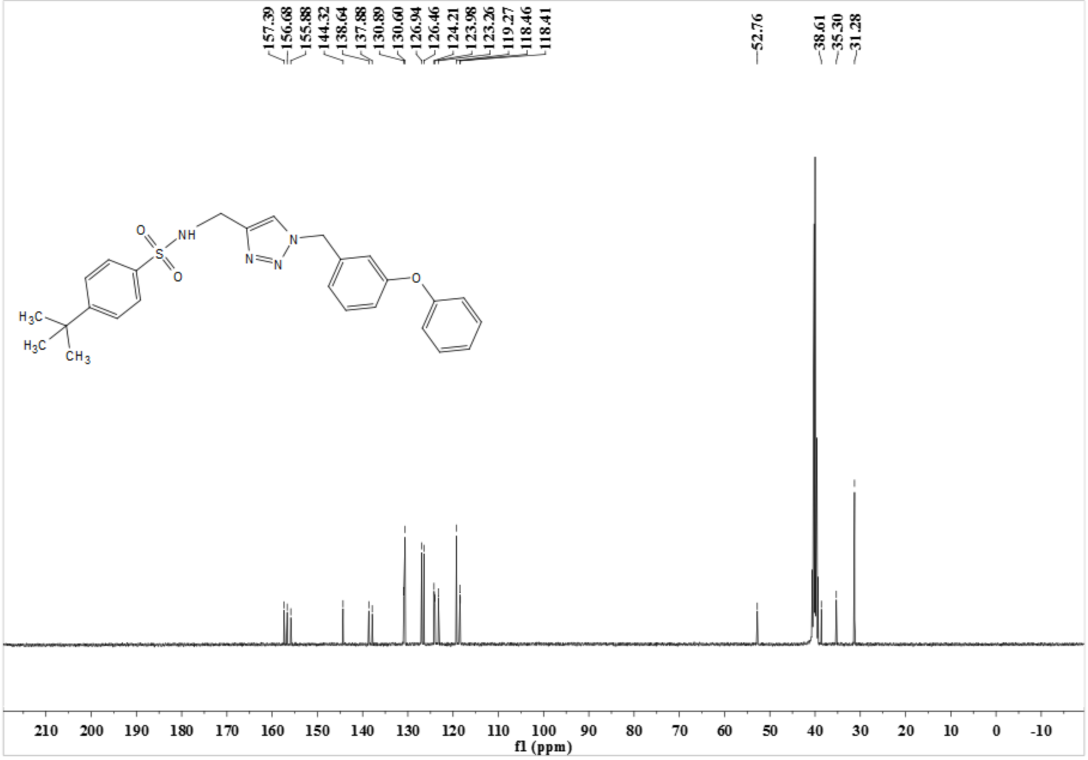


2,4,6-trimethyl-N-((1-(3-phenoxybenzyl)-1H-1,2,3-triazol-4-yl)methyl) benzenesulfonamide (11h)

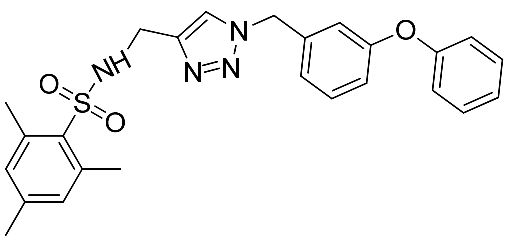

White soild, yield：78.7%, mp: 111-113°C. $^1$H NMR (400 MHz, DMSO) δ 7.95 (s, 1H, NH), 7.70 (s, 1H, Ar), 7.48 – 7.28 (m, 3H, Ar), 7.17 (dd, *J* = 10.6, 4.2 Hz, 1H, Ar), 7.06 – 6.89 (m, 7H, Ar), 5.48 (s, 2H, $PhCH_2$), 4.04 (s, 2H, NH*$CH_2$*), 2.50 (s, 6H, $CH_3$), 2.22 (s, 3H, $CH_3$). $^{13}$C NMR (100 MHz, DMSO) δ 157.34, 156.71, 144.46, 141.83, 138.74, 138.60, 134.94, 132.01, 130.87, 130.61, 124.20, 123.61, 123.26, 119.22, 118.47, 118.43 (Ar), 52.65 ($PhCH_2$), 37.67 (NH*$CH_2$*), 22.98 ($CH_3$), 20.84($CH_3$). HRMS (ESI) calcd for $C_{25}H_{27}N_4O_3S$ $[M+H]^+$: 463.1807, found: 463.1804. IR: 3307, 3269, 1587, 1488, 1326, 1257, 1243, 1154, 736, 657 $cm^{-1}$.

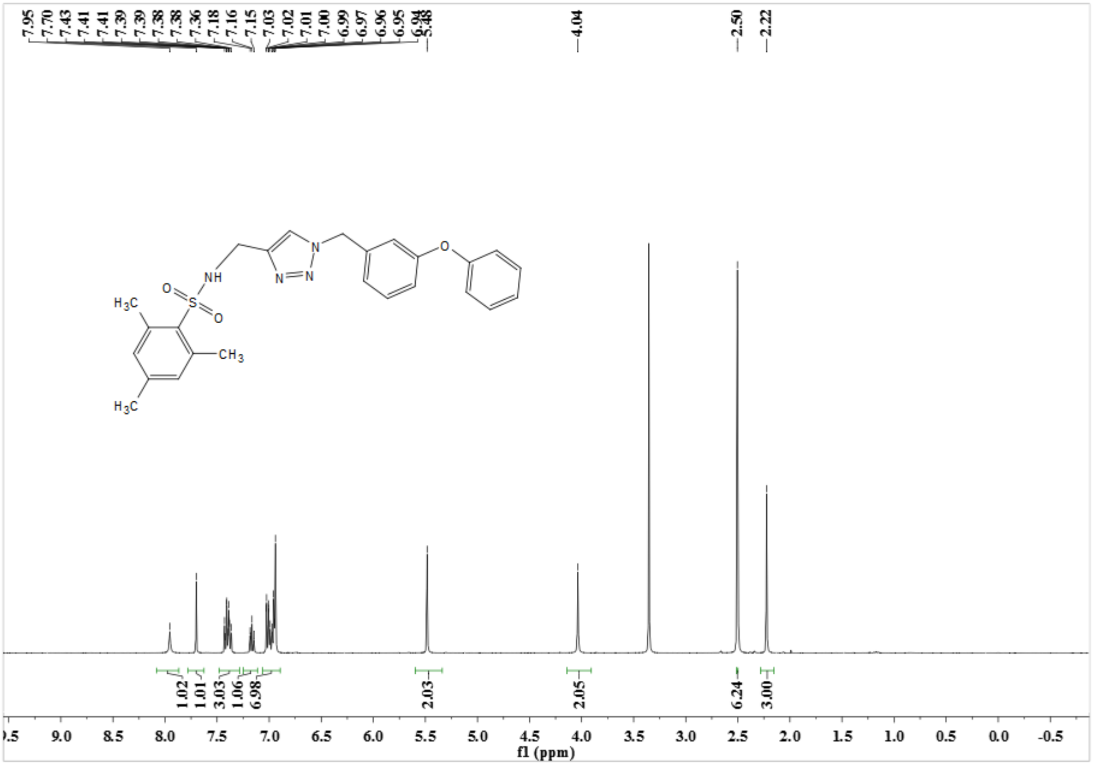

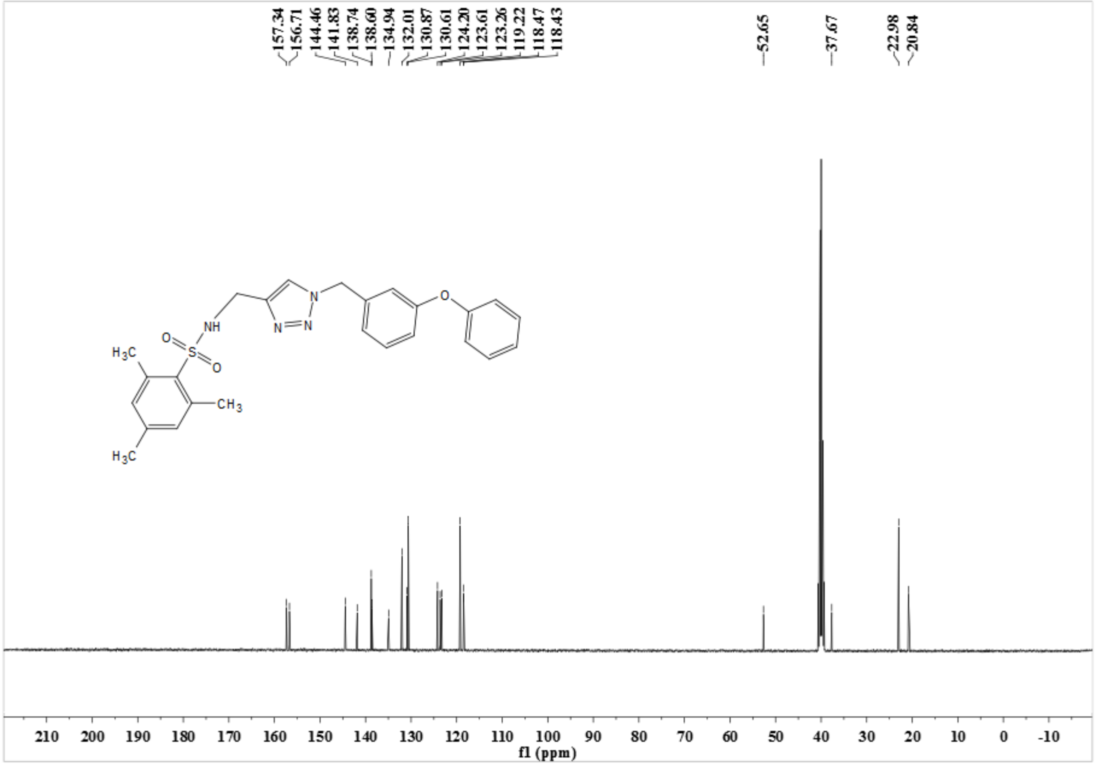

2-chloro-N-((1-(3-phenoxybenzyl)-1H-1,2,3-triazol-4- yl)methyl) benze nesulfonamide (11i)

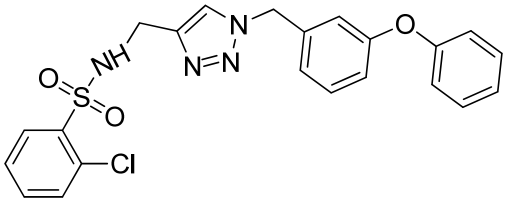

White soild, yield：80.8%, mp: 97-99°C. $^{1}$H NMR (400 MHz, DMSO) δ 8.41 (s, 1H, NH), 7.91 (dd, *J* = 7.8, 1.5 Hz, 1H, Ar), 7.83 (s, 1H, Ar), 7.61 – 7.48 (m, 2H, Ar), 7.48 – 7.32 (m, 4H, Ar), 7.17 (t, *J* = 7.4 Hz, 1H, Ar), 7.10 – 6.89 (m, 5H, Ar), 5.50 (s, 2H, $PhCH_2$), 4.17 (s, 2H, $NHCH_2$). $^{13}$C NMR (100 MHz, DMSO) δ 157.36, 156.70, 144.14, 138.53, 138.49, 134.27, 131.99, 131.05, 130.89, 130.79, 130.62, 127.91, 124.21, 123.83, 123.34, 119.26, 118.54, 118.44 (Ar), 52.67 ($PhCH_2$), 38.31 ($NHCH_2$). HRMS (ESI) calcd for $C_{22}H_{20}ClN_4O_3S$ $[M+H]^+$: 455.0948, found: 455.0945. IR: 3137, 1593, 1489, 1451, 1332, 1253, 1212, 1157, 758, 688, 585 $cm^{-1}$.

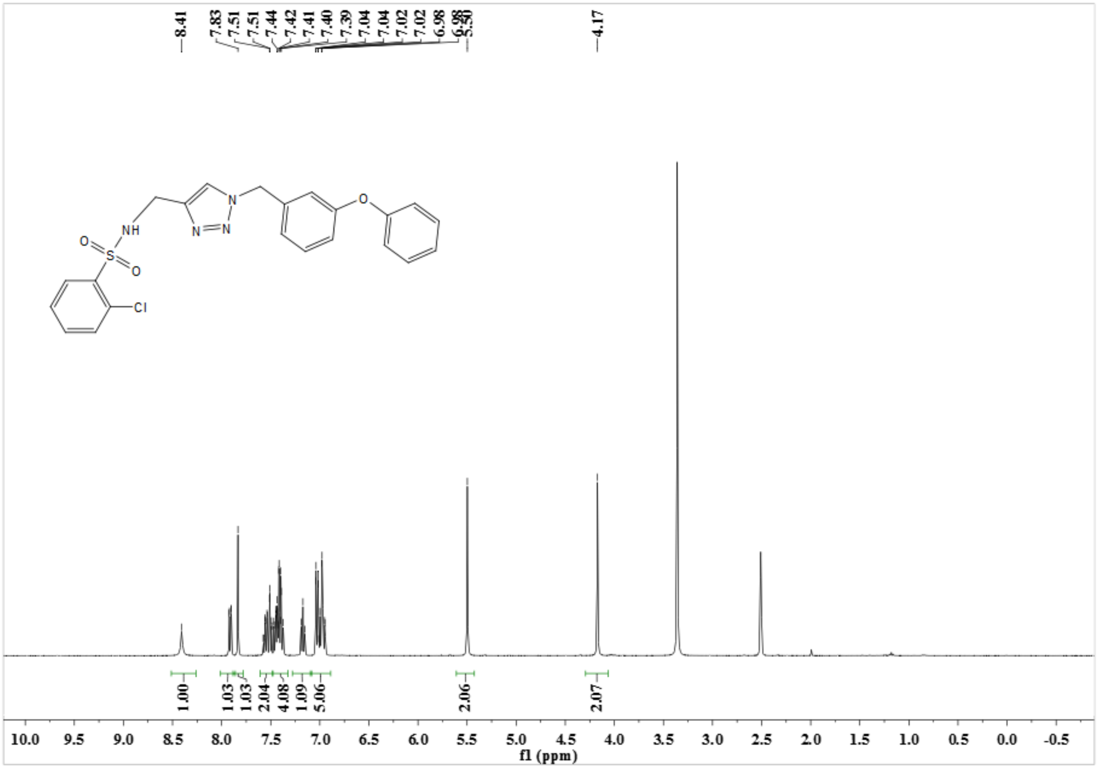


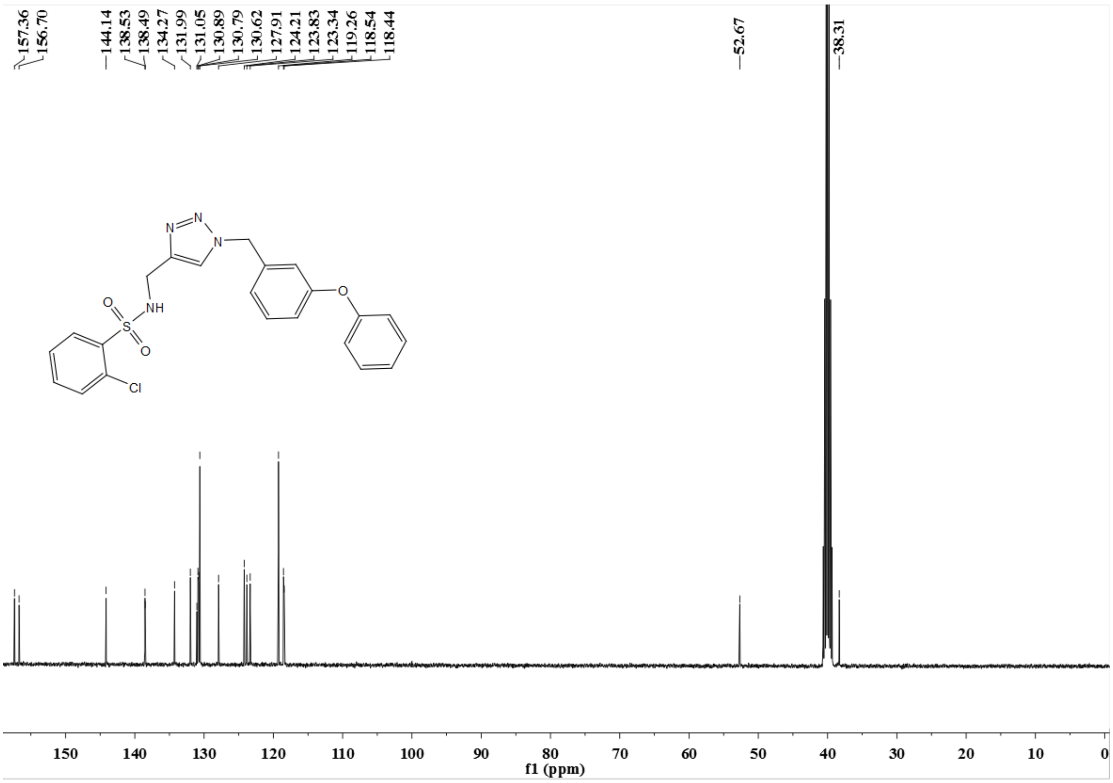


N-((1-(3-cyanobenzyl)-1H-1,2,3-triazol-4-yl)methyl)-4-methylbenzene sulfonamide (11j)

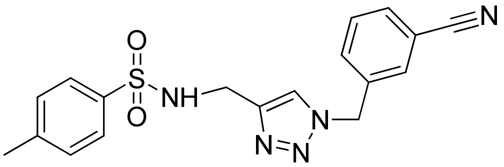

White soild, yield：58.1%, mp: 145-147 ºC. $^{1}$H NMR (400 MHz, DMSO) δ 8.03 (s, 1H, NH), 7.98 (s, 1H, Ar), 7.83 (td, *J* = 4.6, 1.4 Hz, 1H, Ar), 7.77 (s, 1H, Ar), 7.66 (d, *J* = 8.2 Hz, 2H, Ar), 7.60 (d, *J* = 5.0 Hz, 2H, Ar), 7.34 (d, *J* = 8.0 Hz, 2H, Ar), 5.61 (s, 2H, $PhCH_2$), 4.02 (s, 2H, NH$CH_2$), 2.37 (s, 3H, $CH_3$). $^{13}$C NMR (100 MHz, DMSO) δ 143.89, 142.62, 137.55, 137.44, 132.88, 131.95, 131.56, 130.03, 129.50, 126.56, 123.62, 118.40, 111.64 (Ar), 51.70 ($PhCH_2$), 38.05 (NH$CH_2$), 20.93 ($CH_3$). HRMS (ESI) calcd for $C_{18}H_{18}N_5O_2S$ $[M+H]^+$: 368.1181, found: 368.1181. IR: 3265, 2217, 1430, 1323, 1160, 1049, 889, 709, 546 $cm^{-1}$.

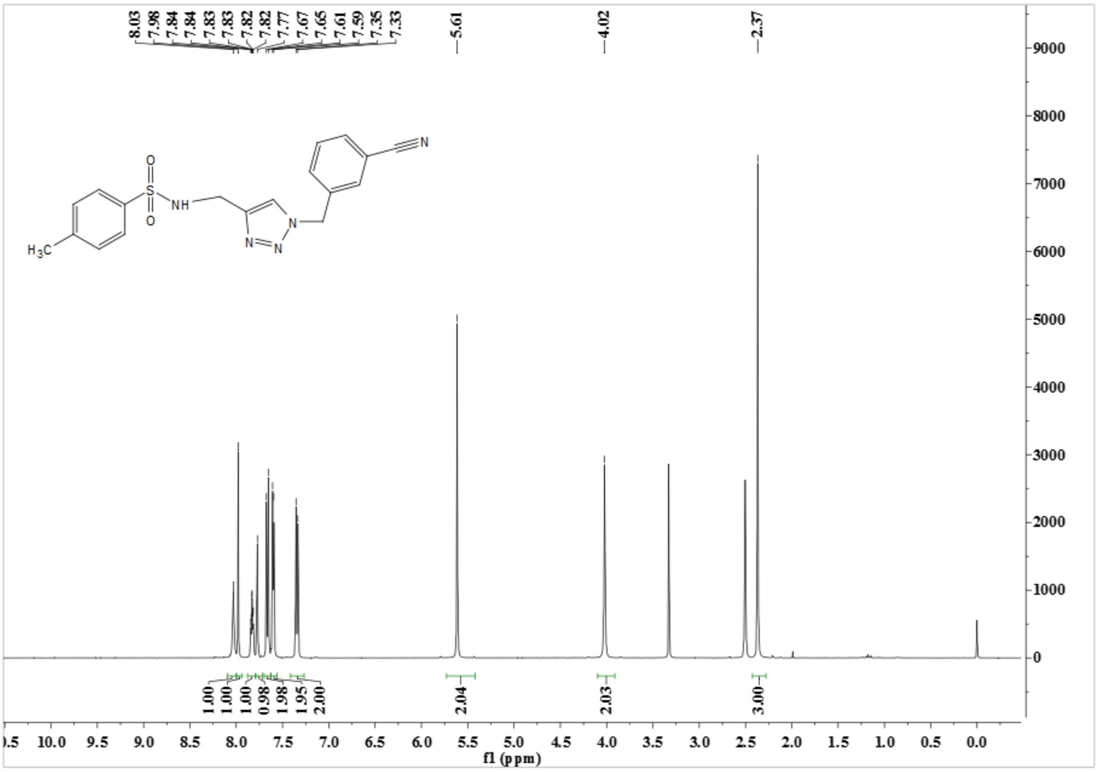

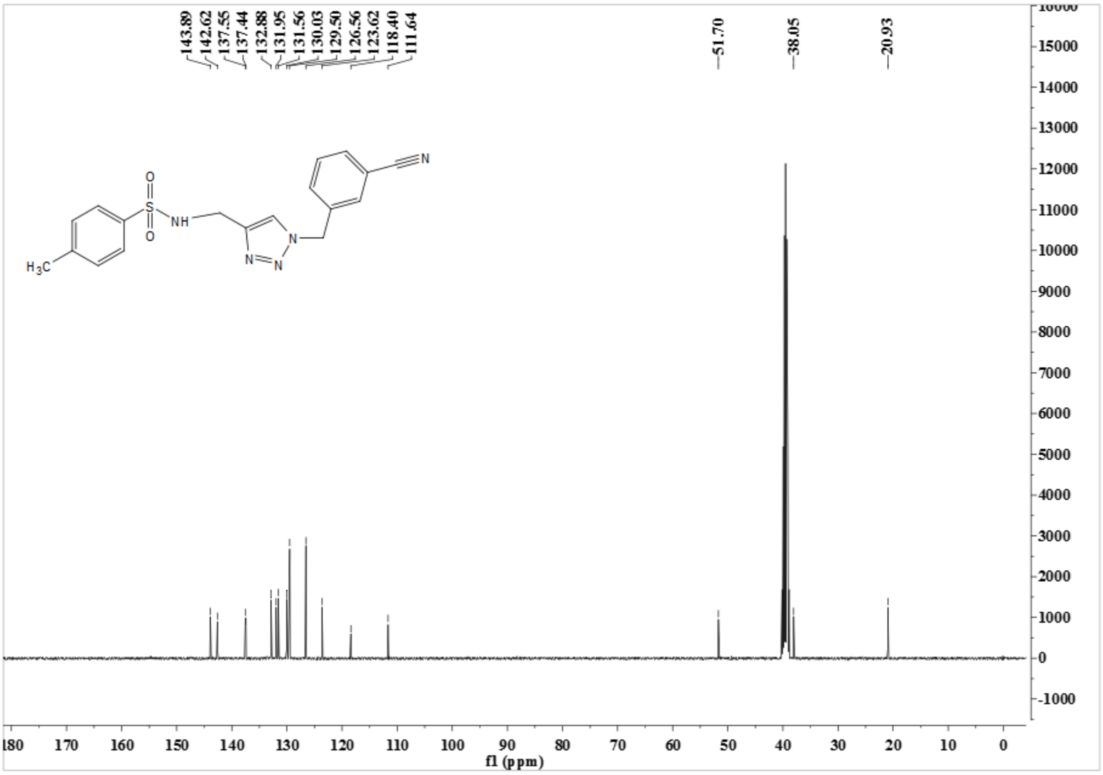


4-methyl-N-((1-(3-methylbenzyl)-1H-1,2,3-triazol-4- yl)methyl)benzenesulfonamide (11k)

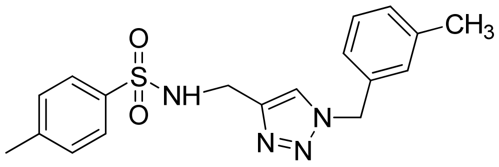


White soild, yield：67.7%, mp: 145-148 ºC. $^1$H NMR (400 MHz, DMSO) δ 8.01 (t, *J* = 6.0 Hz, 1H, NH), 7.87 (s, 1H, Ar), 7.66 (d, *J* = 8.1 Hz, 2H, Ar), 7.34 (d, *J* = 8.0 Hz, 2H, Ar), 7.25 (t, *J* = 7.5 Hz, 1H, Ar), 7.14 (dd, *J* = 15.2, 7.5 Hz, 2H, Ar), 7.06 (d, *J* = 7.6 Hz, 1H, Ar), 5.47 (s, 2H, $PhCH_2$), 4.00 (d, *J* = 6.0 Hz, 2H, NH*$CH_2$*), 2.37 (s, 3H, $CH_3$), 2.29 (s, 3H, $CH_3$). $^{13}$C NMR (100 MHz, DMSO) δ 143.70, 142.61, 137.92, 137.44, 135.83, 129.51, 128.73 , 128.61, 128.55, 126.57 , 125.06 , 123.27 (Ar), 52.69 ($PhCH_2$), 38.09 (NH*$CH_2$*), 20.94 ($CH_3$), 20.89 ($CH_3$). HRMS (ESI) calcd for $C_{18}H_{21}N_4O_2S$ $[M+H]^+$: 357.1388, found: 357.1385. IR: 3258, 3121, 1452, 1325, 1160, 1098, 772, 669, 558 $cm^{-1}$.

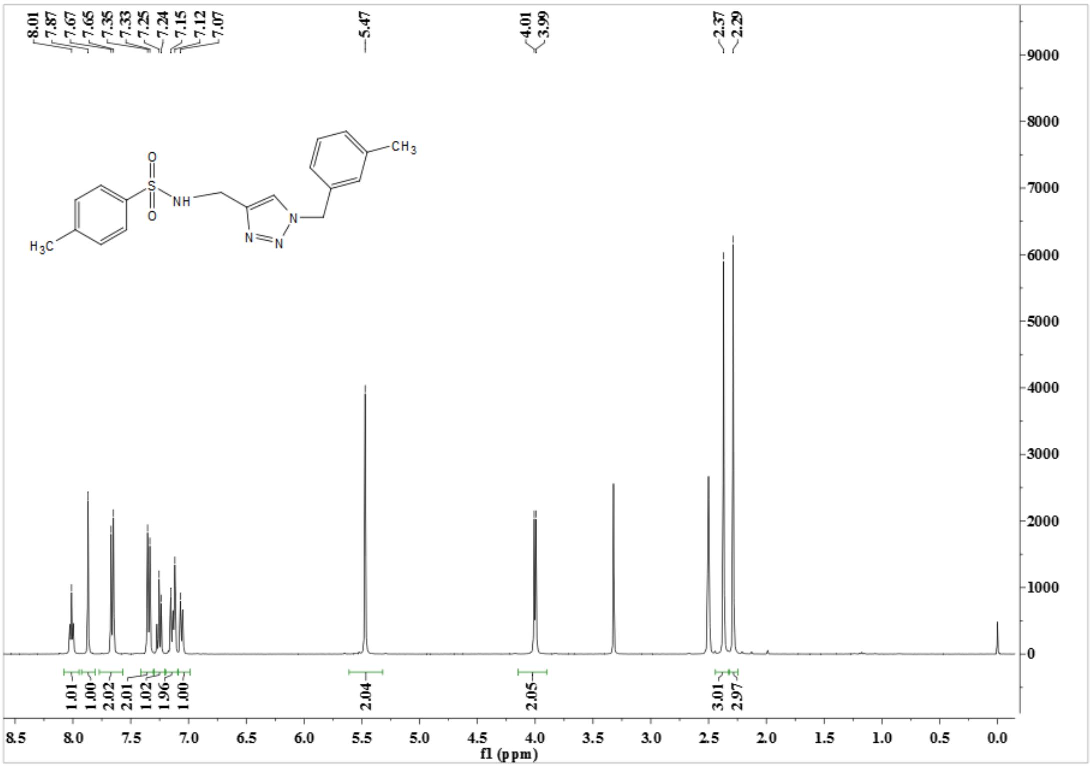

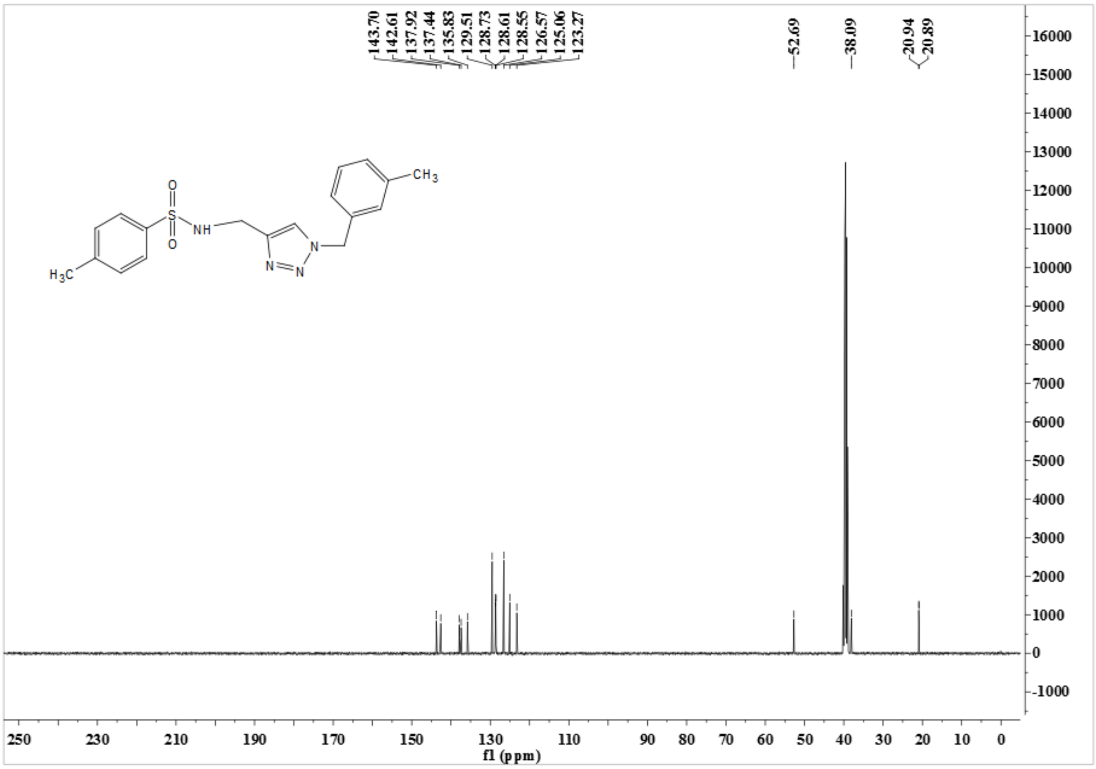

N-((1-(3-methoxybenzyl)-1H-1,2,3-triazol-4-yl)methyl)-4-methylbenzene sulfonamide (11l)

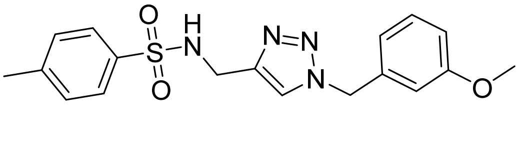

White soild, yield：82.8%, mp: 112-114 ºC. $^{1}$H NMR (400 MHz, DMSO) δ 8.02 (s, 1H, NH), 7.89 (s, 1H, Ar), 7.66 (d, *J* = 8.2 Hz, 2H, Ar), 7.34 (d, *J* = 8.1 Hz, 2H, Ar), 7.28 (t, *J* = 7.9 Hz, 1H, Ar), 6.99 – 6.85 (m, 2H, Ar), 6.82 (d, *J* = 7.6 Hz, 1H, Ar), 5.49 (s, 2H, $PhCH_2$), 4.01 (s, 2H, NH*$CH_2$*), 3.74 (s, 3H, $OCH_3$), 2.37 (s, 3H, $CH_3$). $^{13}$C NMR (100 MHz, DMSO) δ 159.40, 143.72, 142.62, 137.45, 137.38, 129.84, 129.51, 126.56, 123.34, 120.00, 113.79, 113.37 (Ar), 55.08 ($OCH_3$), 52.60 ($PhCH_2$), 38.08 (NH*$CH_2$*), 20.92 ($CH_3$). HRMS (ESI) calcd for $C_{18}H_{21}N_4O_3S$ $[M+H]^+$: 373.1339, found: 373.1334. IR: 3294, 3247, 1600, 1322, 1286, 1265, 1091, 1040, 775, 668, 557 $cm^{-1}$.

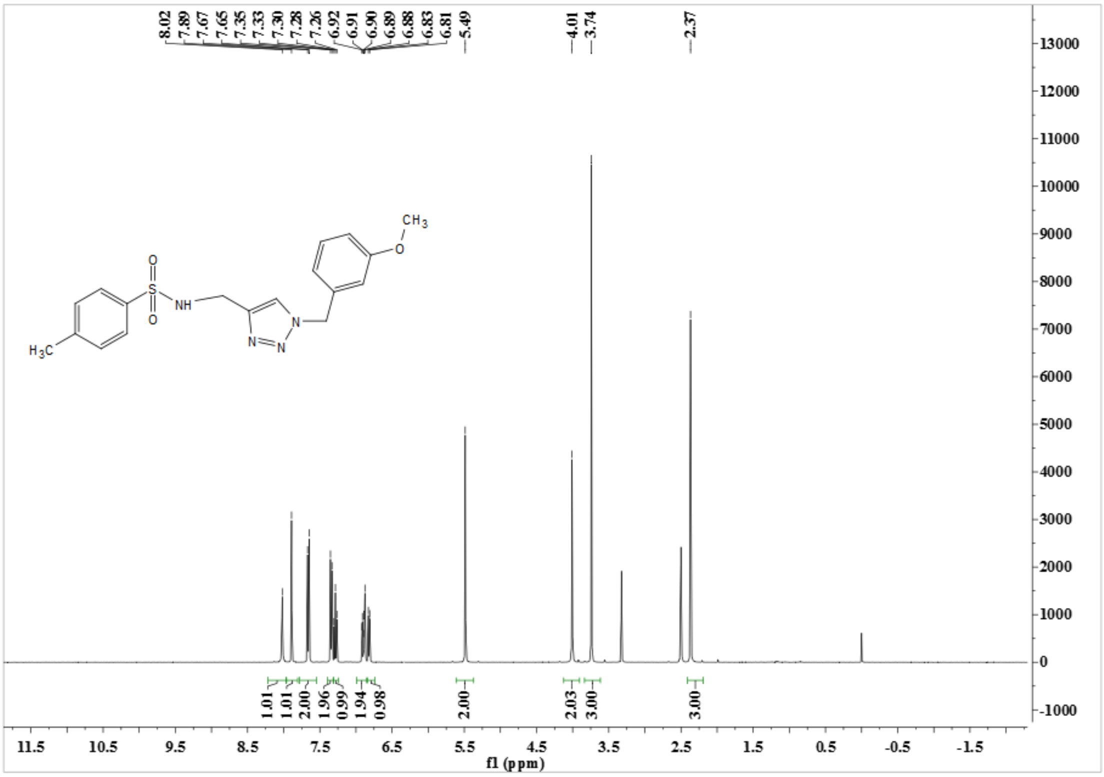

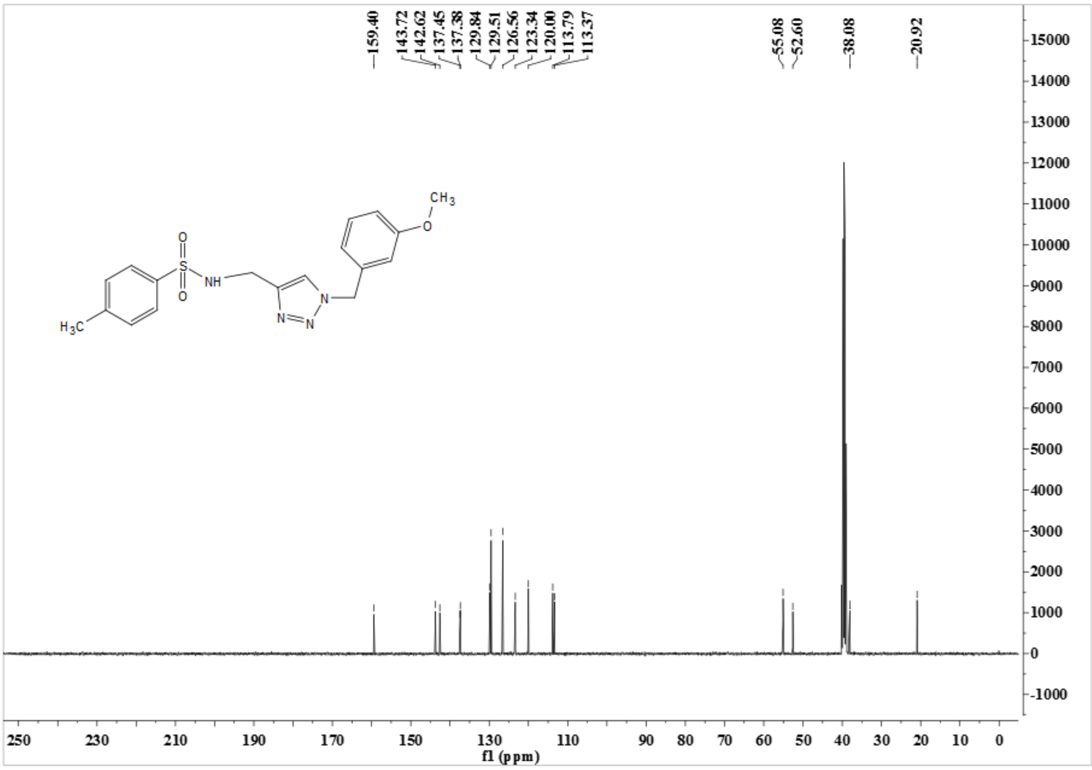

159.40
143.72
142.62
137.45
137.38
129.84
129.51
126.56
123.34
120.00
113.79
113.37
55.08
52.60
38.08
20.92
f1 (ppm)
